# Multifaceted role of one-carbon metabolism on immunometabolic control and growth during pregnancy, lactation and the neonatal period in dairy cattle

**DOI:** 10.1186/s40104-021-00547-5

**Published:** 2021-02-04

**Authors:** Danielle N. Coleman, Abdulrahman S. Alharthi, Yusheng Liang, Matheus Gomes Lopes, Vincenzo Lopreiato, Mario Vailati-Riboni, Juan J. Loor

**Affiliations:** 1 Department of Animal Sciences, Division of Nutritional Sciences, University of Illinois, Urbana, IL 61801 USA; 2 Department of Animal Production, College of Food and Agriculture Sciences, King Saud University, Riyadh, 11451 Saudi Arabia; 3 Department of Animal Sciences, Food and Nutrition, Faculty of Agriculture, Food and Environmental Science, Università Cattolica del Sacro Cuore, 29122 Piacenza, Italy

**Keywords:** Epigenetics, Immune system, Metabolism, Methyl donor, Transition period

## Abstract

Dairy cattle undergo dramatic metabolic, endocrine, physiologic and immune changes during the peripartal period largely due to combined increases in energy requirements for fetal growth and development, milk production, and decreased dry matter intake. The negative nutrient balance that develops results in body fat mobilization, subsequently leading to triacylglycerol (TAG) accumulation in the liver along with reductions in liver function, immune dysfunction and a state of inflammation and oxidative stress. Mobilization of muscle and gluconeogenesis are also enhanced, while intake of vitamins and minerals is decreased, contributing to metabolic and immune dysfunction and oxidative stress. Enhancing post-ruminal supply of methyl donors is one approach that may improve immunometabolism and production synergistically in peripartal cows. At the cellular level, methyl donors (e.g. methionine, choline, betaine and folic acid) interact through one-carbon metabolism to modulate metabolism, immune responses and epigenetic events. By modulating those pathways, methyl donors may help increase the export of very low-density lipoproteins to reduce liver TAG and contribute to antioxidant synthesis to alleviate oxidative stress. Thus, altering one-carbon metabolism through methyl donor supplementation is a viable option to modulate immunometabolism during the peripartal period. This review explores available data on the regulation of one-carbon metabolism pathways in dairy cows in the context of enzyme regulation, cellular sensors and signaling mechanisms that might respond to increased dietary supply of specific methyl donors. Effects of methyl donors beyond the one-carbon metabolism pathways, including production performance, immune cell function, mechanistic target or rapamycin signaling, and fatty acid oxidation will also be highlighted. Furthermore, the effects of body condition and feeding system (total mixed ration vs. pasture) on one-carbon metabolism pathways are explored. Potential effects of methyl donor supply during the pepartum period on dairy calf growth and development also are discussed. Lastly, practical nutritional recommendations related to methyl donor metabolism during the peripartal period are presented. Nutritional management during the peripartal period is a fertile area of research, hence, underscoring the importance for developing a systems understanding of the potential immunometabolic role that dietary methyl donors play during this period to promote health and performance.

## Background

The peripartal period, i.e. the last 3 weeks prepartum through the first 3 weeks postpartum, is characterized by increased inflammation, oxidative stress, adipose tissue mobilization and greater risk of metabolic disorders (e.g. ketosis, fatty liver, milk fever) partly due to reduced dry matter intake (DMI) [1, 2]. Additionally, the exponential growth of the gravid uterus and fetus, followed by the demand of lactation, drive an increase in nutrient requirements during the peripartal period [3]. Decreased DMI leads to a negative nutrient balance (NNB), with a shortfall in nutrient availability for the cow and fetus [4]. The degree and length of time that metabolism and immune responses remain out of balance greatly impact the risk of disease and poor reproduction, subsequently leading to inefficient dairy production.

**Fig. 1:**
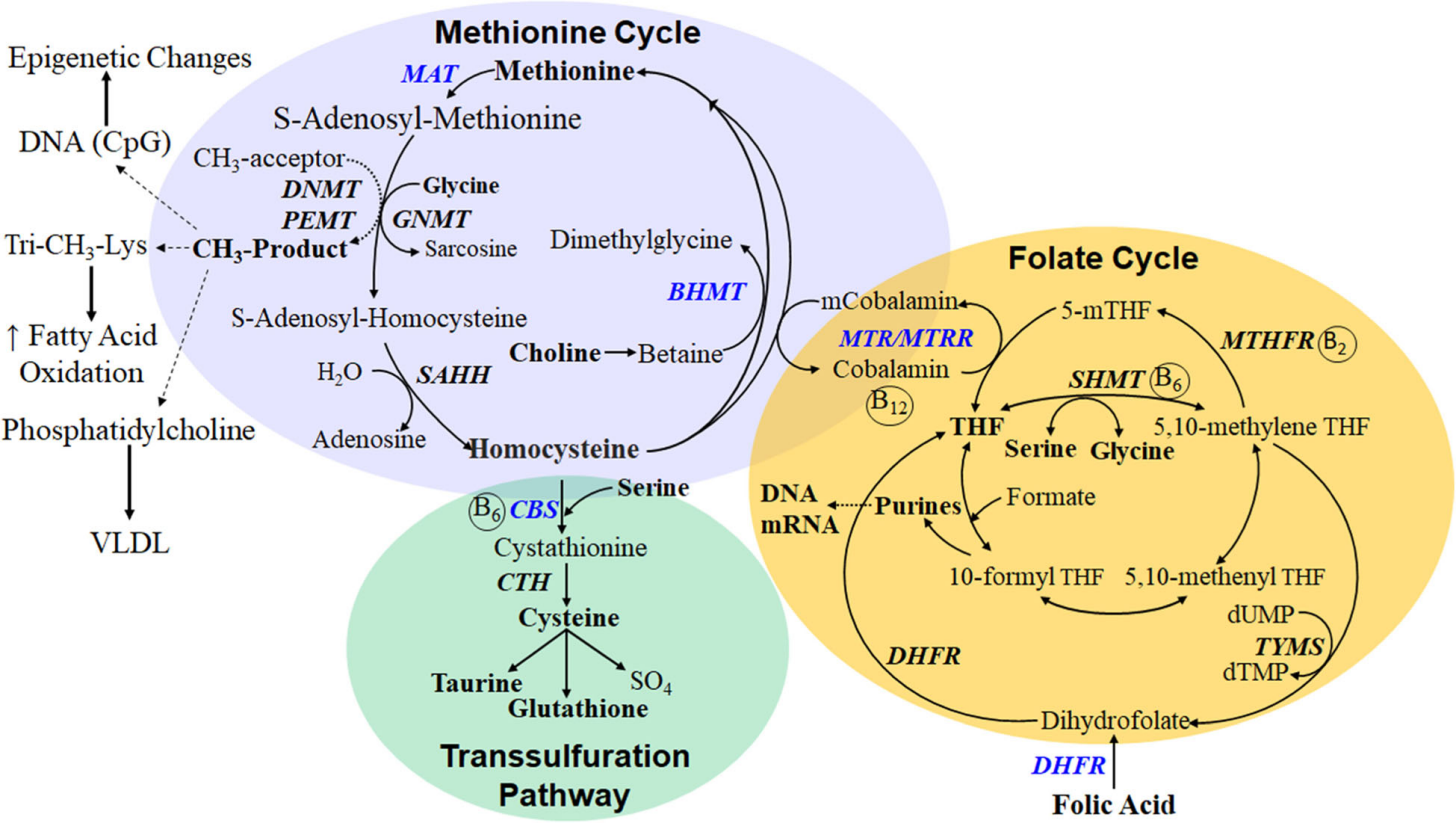
Interrelationships among components of the one-carbon metabolism pathways. 5-mTHF = 5-methyl-tetrahydrofolate; B_2_ = riboflavin; B_6_ = pyridoxal 5′-phosphate; B_12_ = cobalamin; BHMT = betaine homocysteine methyltransferase; CBS = cystathionine beta synthase; CTH = cystathionine gama-lyase; DHFR = dihydrofolate reductase; DNMT = DNA methyltransferase; dUMP = deoxyuridine monophosphate; dTMP = thymidine monophosphate; GNMT = glycine N-methyltransferase; MAT = methionine adenosyltransferase; MTHFR = methylenetetrahydrofolate reductase; MTR = 5-methyltetrahydrofolate-homocysteine methyltransferase; MTRR = 5-methyltetrahydrofolate-homocysteine methyltransferase reductase; PEMT = phosphatidylethanolamine N-methyltransferase; SAHH = *S*-adenosyl-homocysteine hydrolase; SHMT = serine hydroxymethyltransferase; THF = tetrahydrofolate; TYMS = thymidylate synthetase; VLDL = very low-density lipoprotein

Methyl donors (e.g. folate, choline, betaine) serve functional roles throughout the body via their metabolic, epigenetic, and immunomodulatory properties, and share common biochemical pathways, of which one-carbon metabolism has received the most attention (Fig. 1). Despite the fact that major regulatory aspects are relatively well-known in non-ruminants, one-carbon metabolism is not fully understood in ruminants [5, 6]. The unique impact of this pathway in dairy cattle stems from its potential role in embryo development [7], placental function [8], neonatal growth [9], and immunometabolic benefits on the cow [10, 11] during late-pregnancy and early lactation. Besides the periconceptional period (i.e. period from oocyte maturation through early embryo development) [12], given their unique functional roles, the impact of methyl donor nutrition clearly would be multifaceted during late-pregnancy and early lactation, i.e. the peripartal period. Indeed, most research on peripartal cows over the last 2–3 decades has explored these biological interactions to identify mechanisms behind the immunometabolic adaptations that occur, underscoring the need to develop a systemic understanding of the potential immunometabolic role that dietary methyl donors may play during this period.

The main objective of this review is to summarize methyl donor metabolism and physiological adaptations during key stages of the life cycle of dairy cattle in which one-carbon metabolism and methyl donor supply play central roles. As such, the effects of Met, choline, body condition score (BCS) and feeding system on one-carbon metabolism pathways will be discussed. This is followed by discussion of the roles of specific methyl donors beyond the peripartal period and their potential effects at key stages of the life cycle of dairy cattle, including the periconceptional period, transition into lactation, and the neonatal period. Lastly, practical nutritional recommendations related to methyl donor metabolism and the peripartal period are discussed.

### An overview of one-carbon metabolism

One-carbon metabolism encompasses the transfer of carbon atoms in a variety of metabolic reactions and plays a fundamental role in the generation of labile methyl groups [13]. Folate, betaine, methionine (Met), and choline are key nutrients in this pathway. Key interconversions in this pathway include the remethylation of homocysteine to Met using betaine or folate as methyl donors via betaine homocysteine methyltransferase (BHMT) and 5-methyltetrahydrofolate-homocysteine methyltransferase (MTR), respectively (Fig. 1). Choline also participates in Met synthesis through its oxidation to betaine to support BHMT activity. Methionine adenosyltransferase (MAT) converts Met to *S*-adenosyl methionine (SAM), the major cellular methyl donor that in part furnishes a transmethylation reaction catalyzed by phosphatidylethanolamine methyltransferase (PEMT) to generate *S*-adenosylhomocysteine (SAH) and phosphatidylcholine (PC). This is followed by the conversion of SAH to homocysteine in a reversible reaction catalyzed by SAH hydrolase (SAHH). Homocysteine may also enter the transsulfuration pathway, the first reaction of which is used to synthesize cystathionine via the rate-limiting enzyme cystathionine β-synthase (CBS) [14]. Cystathionine can then be used to make cysteine, which is utilized to synthesize the antioxidants glutathione (GSH) and taurine.

### Effect of methionine on one-carbon metabolism pathways

Methionine, a limiting amino acid (AA), participates in one-carbon metabolism [15]. Other than histidine, Met is the only AA for which net uptake by the liver increases soon after parturition [16]. Thus, enhancing post-ruminal Met supply during the peripartal period has received substantial focus during the last decade. Compared with rats, classic studies in sheep demonstrated that hepatic BHMT activity is lower and MTR is greater leading to the first suggestion that MTR plays a crucial role in the remethylation of Met in ruminants. Furthermore, vitamin B_12_, a cofactor of MTR, is produced by the ruminal microorganisms [17], providing a potential link to why MTR is crucial for remethylation of Met. This also points towards the importance of healthy ruminal function to ensure regeneration of Met. Post-ruminal supply of dietary Met is a potential modulator of the enzymes involved in one-carbon metabolism, as increased rumen-protected Met (RPM) supplementation from − 21 d prepartum to 30 d postpartum in dairy cows decreased MTR activity in the liver, which along with increased concentrations of circulating Met [18] implies that MTR is influenced by Met availability. In contrast, an *in vitro* study using polymorphonuclear leukocytes (PMNL) isolated from mid-lactation cows ed that Met supply upregulated mRNA abundance of *MTR* [19]. Furthermore, when the culture medium Lys:Met ratio was increased from a ratio of 3.6:1 to a ratio of 2.4:1, abundance of *MAT2A* (MAT isoform 2A) and *MTR* increased linearly [19]. Overall, available data suggest that MTR activity might be affected by both Met availability and physiological state.

Accumulation of triacylglycerol (TAG) in the liver around parturition has been one of the greatest concerns in the context of dairy cow management and health for over 50 years [20–22]. The first use of high-throughput bovine transcriptome analysis established a link between upregulation of inflammation biomarkers in liver and accumulation of TAG [23], a relationship that was later confirmed through infusion of recombinant bovine tumor necrosis factor-α (TNF-α) [24]. A link between Met supply, liver TAG and very low-density lipoprotein (VLDL) metabolism in the context of susceptibility to ketosis was first discussed in 1968 [20]. However, recent data from large studies in which RPM was fed has underscored the fact that RPM improves liver function in spite of not alleviating accumulation of TAG [11, 25]. Thus, the contribution of Met towards improved liver function is not through reductions in hepatic TAG, but other mechanisms such as reduction of oxidative stress partly as a result of Met metabolism through the one-carbon pathways.

It is well-established in non-ruminants that the antioxidant role of Met arises from its contribution to synthesis of homocysteine and further metabolism via the transsulfuration pathway to the antioxidants taurine and GSH [26]. Of note, emerging evidence suggests that this also applies to ruminants. For example, cows  fed RPM had greater mRNA abundance of *SAHH, MTR,* superoxide dismutase (*SOD1*), glutamate-cysteine ligase catalytic subunit (*GCLC*), and DNA methyltransferase (*DNMT*) 3 in the liver, suggesting alterations in flux through one-carbon metabolism pathways. Furthermore, feeding RPM increased circulating plasma concentrations of cystathionine, cystine, homocysteine, and taurine, suggesting increased flux through the transsulfuration pathway [27, 28]. Although not every step of these pathways has been examined in the context of mechanisms, the consistent responses in terms of decreased plasma reactive oxygen metabolite concentrations, coupled with increased concentrations of ferric-reducing antioxidant power, β-carotene, tocopherol, and total and reduced GSH (also in liver tissue) in response to feeding RPM support the antioxidant role of Met [25, 29]. In addition to these systemic biomarkers, there are reports of increased plasma total antioxidant capacity, glutathione peroxidase (GPX) activity and vitamin E concentrations when post-ruminal RPM supply increased (76), suggesting that RPM plays a critical role in regulating oxidative stress in peripartal cows.

At least in non-ruminants, CBS is allosterically activated by SAM. Activity of CBS may also be regulated by Met supply, as greater dietary Met supply typically leads to greater hepatic CBS activity in non-ruminants [30]. An increase in oxidant status also increases CBS activity to promote GSH or taurine synthesis [31, 32]. In vitro, adding Met to the culture medium enhanced mRNA and protein abundance of CBS, as well as MAT1A (MAT isoform 1A) and PEMT in bovine primary hepatocytes. This suggested that the increase in MAT1A abundance may help to enhance SAM production and flux through the transsulfuration pathway. Furthermore, enhanced post-ruminal supply of Met from 21 d prepartum through 30 d postpartum led to reduced oxidative stress along with increased *CBS* abundance and CBS activity in dairy cows [27, 28]. As mentioned previously, RPM-fed cows had greater concentrations of GSH in plasma and liver tissue [29, 33]. Thus, available data suggest that improvements in liver function and reductions in oxidative stress in response to RPM supply in dairy cows might at least be partly due to enhanced GSH synthesis via one-carbon metabolism.

### Effect of choline on one-carbon metabolism pathways

Choline plays a central role in one-carbon metabolism where it is utilized in the remethylation of homocysteine to Met, DNA and protein methylation, and the production of PC, especially in non-ruminants capable of absorbing it directly from the gut. The latter feature of choline metabolism has been of particular interest for a number of decades [21] especially upon recognition that, unlike non-ruminants, differences in activity of MTR and BHMT led to the conclusion that ruminants “spared” choline for unique functions such as methylation reactions [34]. As with Met, choline supplementation has also been associated with changes in activity and abundance of enzymes in one-carbon metabolism. In a study by Zhou et al., rumen-protected choline (RPC) supply from d − 21 through 30 around parturition did not alter activity of BHMT, but overall MTR activity was lower with RPC. Despite the lack of difference in BHMT activity, it is possible that Met synthesis was still enhanced with choline supply, as the mRNA abundance of *MAT1A* and dimethylglycine dehydrogenase (dimethylglycine is produced when betaine is metabolized via BHMT) were increased by RPC compared with control cows [27]. Furthermore, enhancing post-ruminal supply of choline via abomasal infusion during a feed restriction-induced NNB linearly increased BHMT activity and tended to linearly increase MTR activity, which was in agreement with *in vitro* increases in hepatocyte mRNA abundance of *BHMT* and *MTR* with choline [35, 36]. Those changes in enzyme activity were associated with increases in liver and plasma Met, underscoring the importance of choline and BHMT for *de novo* synthesis of Met during periods of NNB. Lastly, the role of vitamin B_12_ as a cofactor for MTR may also play a role in driving the importance of BHMT rather than MTR during periods of NNB; with limited intakes, ruminal production of vitamin B_12_ would be decreased, leading to a potential shortfall in its availability for MTR. Overall, cows that are unable to consume enough Met from the diet during NNB could alleviate a shortfall of this AA through choline oxidation.

**Fig. 2:**
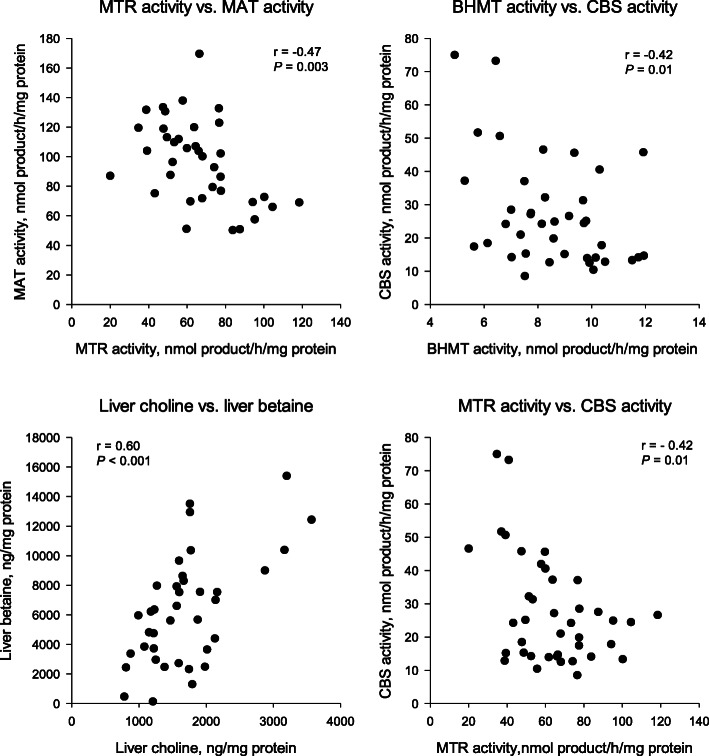
Pearson correlations between hepatic cystathionine β-synthase (CBS) and betaine homocysteine methyltransferase (BHMT) activity, CBS and 5-methyltetrahydrofolaare-homocysteine methyltransferase (MTR) activity, MTR and methionine adenosyltransferase (MAT) activity and liver choline and betaine concentrations in Holstein cows fed to 60% of their net energy for lactation requirements and receiving abomasal infusions of 0, 6.25, 12.5 or 25 g/d choline ion for 4 d [37, 38]

In the transsulfuration pathway, CBS activity was not altered by RPC supply during the peripartal period in the study by Zhou et al. [27]. However, Coleman et al. observed a linear decrease in CBS activity with enhanced post-ruminal choline supply during conditions of NNB, implying that choline supply may alter CBS activity. Furthermore, in that study there was a negative correlation between CBS and both MTR (*r* = − 0.42; *P* = 0.01; Fig. 2) and BHMT (*r* = − 0.42; *P* = 0.01; Fig. 2), indicating that maintenance of Met synthesis during NNB is critical during periods of NNB and seems to supersede the transsulfuration pathway (note, these correlations are not published and were conducted as part of this review). In rats, the K_m_ of CBS from homocysteine is estimated at 4.8 mmol/L and for serine, a cofactor, at 2.0 to 3.0 mmol/L. [39] Thus, at high concentrations of homocysteine, the excess will be removed by activating the transsulfuration pathway through changes in the activity of CBS [39]. As such, the decrease in CBS activity in the study by Coleman et al. [37] likely corresponded with the observed increases in MTR and BHMT activity. Despite the fact that SAM production was likely increased, it was preferntially being used to synthesize carnitine to support fatty acid oxidation as will be discussed later, rather than to activate CBS. Thus, choline supply during periods of NNB alters one-carbon metabolism pathways to promote Met synthesis during this time when Met intake (or its supply from microbial protein), as well as vitamin B_12_ synthesis, is limited.

### Effect of body condition and prepartal energy allowance on one-carbon metabolism pathways

The transition period is one of the most-important stages of the lactation cycle in dairy cattle, characterized by significant metabolic, oxidant status, and immune challenges [22, 40, 41]. Because failure to adequately meet these challenges can compromise production, induce metabolic diseases, and increase rates of culling in early lactation [42], management of the transition cow remains a focal point for dairy producers. Traditional management provides dry cows with a high-fiber/low-energy density ration, increasing energy density and reducing fiber content in the 3–4 weeks. prior to parturition. This practice originated from the recommendations by Boutflour [43]. However, studies conducted in the last decades have demonstrated that prepartum overfeeding of energy often leads to a wrecked transition [44–52].

Despite the fact that cow adiposity (measured through body condition score, BCS, a qualitative measurement) plays an important role in the metabolic response of the animal to lactation and its level is regulated through nutrition, cows with different level of adiposity are generally managed similarly during the prepartum period. The connection between prepartal nutrition and BCS is further strengthened by the fact that similar negative responses to overnutrition also have been observed when cows reached parturition at extreme levels (e.g., too high or too low) of BCS [45, 53–59].

Management of both nutrition and adiposity has proven effective in counteracting the most negative effects of transient metabolic and inflammatory status during the transition period [60, 61]. Due to the tight link between metabolic and redox status [40], and the previously established importance of one-carbon metabolism in supplying the systemic antioxidant pool, a connection between prepartal BCS or nutritional management and the transition period Met supply and one-carbon metabolism is not far-fetched.

No data are available in confinement systems regarding the outcomes of BCS modulation on the status of the Met cycle. While looking, instead, at the effects of a higher-energy close-up diet, Vailati-Riboni et al. [62] detected only few changes at the mRNA level with cows receiving a higher-energy diet prepartum (e.g., 1.54 Mcal/kg DM) having greater *DNMT3A* and lower *MTR* hepatic abundance compared with cows fed a higher-fiber diet (e.g., 1.24 Mcal/kg DM). These changes suggested a greater degree of utilization and a lower capacity to regenerate Met, which could place a strain on the available cellular pool of this essential AA.

When investigating the effect of prepartum BCS and energy level in a pasture-based system (i.e. m^2^/cow), Vailati-Riboni et al. [63] identified hepatic transcriptomic changes related to the Met cycle (e.g. folate biosynthesis, one-carbon pool by folate, vitamin B_6_ metabolism) when underconditioned cows (e.g., 2.5 on a 4 point scale) were allowed a higher pasture area prepartum, thus, increasing their overall energy supply to approximately 110% of estimated ME requirements (calculated post trial via equations using blood metabolites). The changes detected in these three pathways seemed to indicate a greater flux through the one-carbon metabolism pathways, with a potential increase in the availability of Met and antioxidants. Together with the increase in activation of taurine and hypotaurine metabolism during early lactation in those cows, these results from Vailati-Riboni et al. [63] suggested a link between AA metabolism and energy intake, together with a beneficial alteration of Met metabolism via prepartal energy intake manipulation.

Manipulation of both prepartum cow adiposity and overall energy intake via pasture allocation also had a marked impact on abundance and activity of enzymes involved in one-carbon metabolism (e.g. MTR and BHMT), transsulfuration pathway (e.g. CBS), and Krebs cycle [64], suggesting that greater flux of dietary methyl donors through these pathways in optimally-conditioned cows (e.g., 3–3.25 BCS on a 4 point scale) and in cows that were feed-restricted prepartum to 75% of their ME requirements. Furthermore, when evaluating the interaction between prepartum adiposity and energy allowance, greater concentrations of metabolites in the one-carbon metabolism, Met and folate cycles measured via a targeted metabolomics approach indicated a consistently greater flux throughout these and their related pathways in feed-restricted, optimally-conditioned cows. These results confirmed the authors’ previous hypothesis and recommendations regarding the use of separate prepartal dietary energy levels based on BCS at dry off. Thanks to the observed changes, authors speculated that optimally-conditioned cows, with a BCS between 3 and 3.25 on a 4-point scale, should be slightly feed-restricted (~ 90% of ME requirements) during the close up period, while thinner cows (e.g., BCS of 2.5 on a 4-point scale) should receive a slight increase in ME (~ 110% of estimated requirements) in the same period, to better withstand the metabolic, redox, and inflammatory challenges of the transition period.

### Effect of feeding system on one-carbon metabolism pathways

The advantage of a pasture-based dairy system is centred around the low cost of pasture compared with more controlled total mixed ration (TMR) systems, based on conserved forages and concentrates. For their profitability, however, grazing systems depend on high levels of pasture production, and on the efficiency with which cows are able to harvest it. Regarding peripartal dairy cows, the research focus has been on energy nutrition [65], as intake of energy in pasture systems is a major limiting factor for milk production [66]. On the other hand, protein supply in pasture-based diets, both in terms of quantity and quality, has always been considered adequate [66, 67]. With regards to specific AA supply, lysine and Met supply is a well-known limiting factor for milk production in high-producing confinement systems, while in pasture-based systems, their supplementation has not proven effective [67, 68]. These AA limitations will, in fact, only affect production if there are no other first-limiting nutrients. Since energy intake is the first-limiting factor for milk production in grazing systems, supplementation of high-quality pasture would first need to correct the deficiency in energy rather than AA supply [65].

In dairy cows, hepatic MTR and BHMT activity also changes throughout the peripartal period whether cows are managed in a TMR- or grazing-system. Activity of both MTR and BHMT increased in the postpartum compared with prepartum in a TMR-based system [27, 28]. However, in a pasture-based system, BHMT activity followed a similar trend while MTR did not, and postpartal MTR activity decreased over time reaching half the levels of those reported for TMR-fed cows [64]. Zhou et al. [27] in a TMR-system reported a 130% and 26% increase inhepatic activity of BHMT and MTR, respectively, around parturition, and a similar response (+ 72%) in BHMT hepatic abundance, but no changes in MTR abundance. In addition, they reported a 20% and 13% increase in hepatic CBS activity and abundance, respectively, postpartum, when compared with prepartum. Thus, compared with CBS activity, the greater increase in MTR and BHMT activity postpartum highlighted a preference for Met synthesis rather than for the transsulfuration pathway in transition cows reared in intense TMR-based confinement systems. Compared with their counterparts in confinement feeding systems, pasture-based systems utilize cows genetically selected with more emphasis on fertility and body condition score, and less on milk production. However, despite the lower milk production, the physiological and immunological dysfunction during the transition period in grazing dairy cows appears to be of similar magnitude to the higher-producing TMR-fed counterparts [61]. This said, recent data looking at these mechanisms in pasture-raised dairy cows appear to underscore fundamental differences between the two systems in the one-carbon and transsulfuration pathway activity [64]. For instance, BHMT activity and mRNA abundance were the only parameters in pasture cows that resembled the trends reported in confinement systems [27, 64]. However, activity values, despite being similar prepartum, were half of those identified previously in TMR-fed cows [27]. Furthermore, despite the increase in concentrations of metabolites supplying substrates to MTR (e.g. folate, glycine, serine), its activity decreased postpartum [64]; this is contrary to what was reported in TMR-fed cows [27]. Furthermore, the 2- to 3-fold greater MTR activity prepartum and early postpartum in pasture-fed compared with TMR-fed cows suggested a more critical role of this enzyme in grazing cows probably due to the high concentration of folate in their diet, a precursor of 5-methyl-tetrahydrofolate and substrate of MTR, which is abundant in green leafy forage [69].

Concerning the transsulfuration pathway, the fact that homocysteine was undetectable, and that greater concentrations of its metabolites (e.g. cystathionine, hypotaurine, serine) were detected in liver tissue postpartum suggested increased flux through this pathway in pasture-fed dairy cows [64]. However, contrary to what was reported in TMR-based systems [27], activity and abundance of CBS decreased postpartum [64]. While it is unclear how metabolites in the transsulfuration pathway increased without a change in CBS activity, it is possible that there was feedback inhibition of this enzyme by intermediate metabolites in the pathway. Furthermore, CBS activity in grazing cows was close to half of those in higher-producing TMR-fed cows. Thus, this increase in homocysteine-related metabolites across the peripartal period could be linked to the greater need for antioxidant synthesis to combat oxidative stress associated with NNB [1, 2]; however, more work is needed to verify the mechanisms behind increases in transsulfuration pathway intermediate metabolites.

Despite an extensive review of the literature concluding that grazing cows undergo a similar degree of peripartal physiological dysfunction than high-producing animals in TMR-systems [70], the substantial differences in BHMT, MTR, and CBS activity suggest a different load on the metabolic activity of the one-carbon metabolism and transsulfuration pathways. The unique trends between systems during the transition period might be explained by the difference in demand for lactation (e.g. lower milk production) and in nutrient intake. Overall, the precise mechanisms behind the differences between feeding systems are not clear, but likely involve total nutrient supply (i.e. greater in TMR-systems), level of milk production, and/or nutrient demands on pasture versus TMR.

### Correlations between activity of one-carbon metabolism enzymes and production performance and one-carbon metabolism substrates in the liver

To our knowledge, the sole data on hepatic activity of key one-carbon metabolism enzymes across the peripartal period (TMR-fed and pasture-based systems) have been reported by our laboratory. Thus, to further investigate how BHMT, MTR and CBS are regulated in peripartal cows, for this review we conducted a correlation analysis of data from three of our studies [27, 28, 64] (Table 1). In the study by Zhou et al., cows were fed a TMR with or without RPM at 0.08% DM intake (DMI) for 21 d prepartum through 30 d postpartum. In the second study with TMR-fed cows, Vailati-Riboni et al. fed diets with or without RPM at 0.10% DMI from 28 d prepartum through 60 d postpartum. In the last study with pasture-fed cows, a 2 × 2 factorial design of BCS and energy level during the periparturient period was used: 2 prepartum BCS categories [4.0 (thin, BCS 4) and 5.0 (optimal, BCS 5); 10-point scale] and 2 levels of energy intake during the 3 weeks. preceding calving (75% or 125% of estimated requirements) obtained via allowance (m^2^/cow) of fresh pasture composed of mostly perennial ryegrass and white clover.

**Table 1:** Pearson correlation coefficients among hepatic cystathionine β-synthase (CBS), betaine homocysteine methyltransferase (BHMT) and 5-methyltetrahydrofolate-homocysteine methyltransferase (MTR) activity, liver triacylglycerol (TAG), dry matter intake (DMI), milk yield (MY), plasma methionine (Met), liver glutathione (GSH) and liver Met in periparal dairy cows^a^

Variable	CBS activity	MTR activity	BHMT activity	Liver TAG	DMI	MY	Plasma Met	Total liver GSH	Liver Met
MTR activity	−0.17^*^								
BHMT activity	0.50^*^	−0.002							
Liver TAG	0.31^*^	0.09	0.29^*^						
DMI	0.29^*^	−0.28^*^	−0.12^+^	0.16^*^					
MY	0.66*	−0.36^*^	0.13^+^	0.05	0.78^*^				
Plasma Met	0.12^+^	−0.31^*^	−0.07	−0.09	0.26^*^	0.24^*^			
Total liver GSH	−0.06	0.10	0.05	−0.06	−0.06	− 0.06	−0.21^*^		
Liver Met	0.83^*^	−0.35^*^	0.84^*^	0.20	0.40^*^	0.88^*^	−0.13	−0.14	
Liver betaine	0.83^*^	−0.34^*^	0.84^*^	0.27	−0.11	0.87^*^	0.08	0.28^+^	0.97^*^

^a^Data from three transition cow studies were used for analysis [27, 28, 64]. In the study by Zhou et al. cows were fed a total mixed ration with or without rumen-protected Met (RPM) at 0.08% DM for 21 d prepartum and 30 d postpartum. Vailati-Riboni et al. fed diets with or without RPM at 0.10% DM for 28 d prepartum and 60 d postpartum. In the last study, a 2 × 2 factorial design of body condition score (BCS) and energy level during the peripartal period was used: 2 prepartum BCS categories [4.0 (thin, BCS 4) and 5.0 (optimal, BCS 5); 10-point scale] and 2 levels of energy intake during the 3 weeks. preceding calving (75% or 125% of estimated requirements) obtained via allowance (m^2^/cow) of fresh pasture composed of mostly perennial ryegrass and white clover. Due to the large range and distribution of the metabolomics data variables for liver Met and betaine, they were log-transformed. Values reported in the graphs are log values

^*^P ≤ 0.05; ^+^*P* ≤ 0.10

**Fig. 3:**
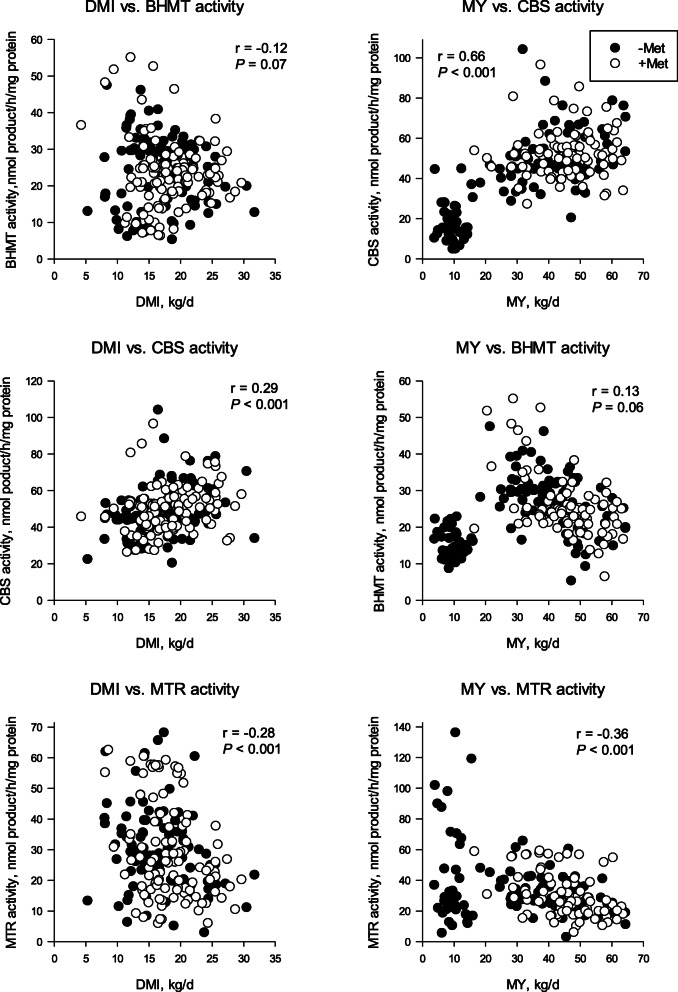
Pearson correlations between hepatic cystathionine β-synthase (CBS), betaine homocysteine methyltransferase (BHMT) and 5-methyltetrahydrofolaare-homocysteine methyltransferase (MTR) activity with dry matter intake (DMI) and milk yield (MY) from 3 studies [11, 64, 71]. In the studies by Zhou et al. [27, 71] cows were fed a total mixed ration with or without rumen-protected Met (RPM) at 0.08% DM for 21 d prepartum and 30 d postpartum. Batistel et al. [11] fed diets with or without RPM at 0.10% DM for 28 d prepartum and 60 d postpartum. Enzyme and metabolite data from this study was reported by Vailati-Riboni et al. [28]. In the last study, a 2 × 2 factorial design of body condition score (BCS) and energy level during the peripartal period was used: 2 prepartum BCS categories [4.0 (thin, BCS 4) and 5.0 (optimal, BCS 5); 10-point scale [72]] and 2 levels of energy intake during the 3 weeks preceding calving (75% or 125% of estimated requirements) obtained via allowance (m^2^/cow) of fresh pasture composed of mostly perennial ryegrass and white clover [64]. Data in the graphs are split between cows receiving Met and those that did not receive Met

While DMI was negatively correlated with MTR activity (*r* = − 0.28; *P* < 0.001; Fig. 3) and tended to be with BHMT (*r* = − 0.12; *P* = 0.07; Fig. 3), the correlations were not strong. The same negative correlation was observed between milk yield and MTR (*r* = − 0.36; *P* < 0.001; Fig. 3) and BHMT (*r* = 0.13. *P* = 0.06; Fig. 3). Thus, while increased DMI and milk yield may be associated with a decrease in remethylation of homocysteine to Met, they do not seem to be the main drivers regulating activity of MTR and BHMT. In contrast, a positive correlation was observed between both DMI (*r* = 0.29; *P* < 0.001; Fig. 3) and milk yield (*r* = 0.66; *P* < 0.001; Fig. 3) with CBS activity. The strong correlation between CBS and milk yield is likely related to a reduction in oxidative stress in the liver due to greater flux through the transsulfuration pathway, reiterating the importance of promoting liver function in the peripartal period to enhance milk production.

**Fig. 4:**
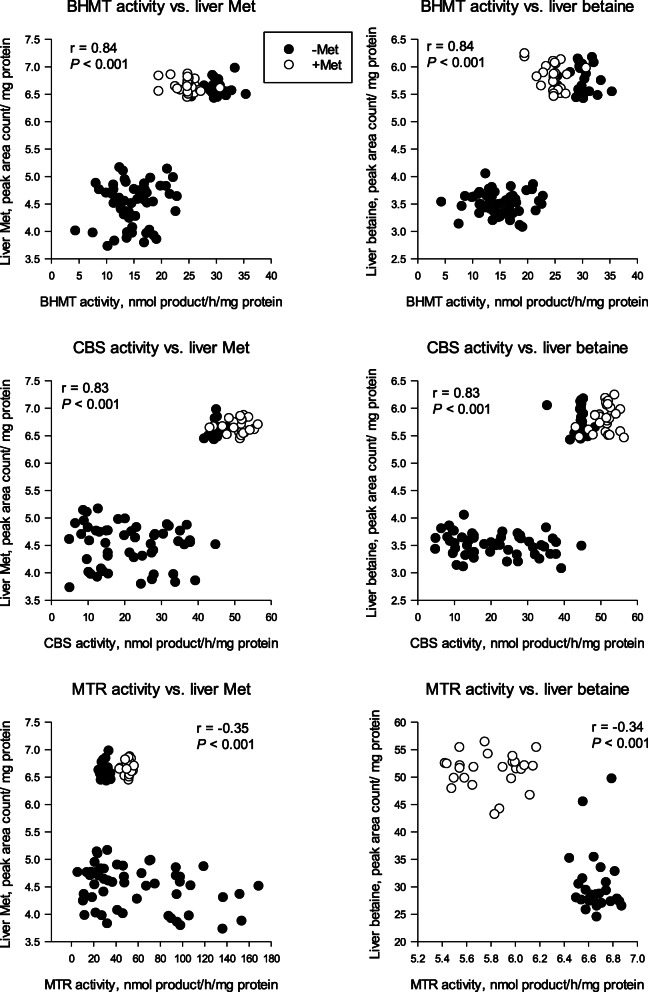
Pearson correlations among hepatic cystathionine β-synthase (CBS), betaine homocysteine methyltransferase (BHMT) and 5-methyltetrahydrofolaare-homocysteine methyltransferase (MTR) activity with concentrations of liver methionine (Met) and liver betaine from 2 studies [28, 64]. In the study first study Vailati-Riboni et al. [28] fed diets with or without RPM at 0.10% DM for 28 d prepartum and 60 d postpartum. In the last study, a 2 × 2 factorial design of body condition score (BCS) and energy level during the periparturient period as used: 2 prepartum BCS categories [4.0 (thin, BCS 4) and 5.0 (optimal, BCS 5); 10-point scale [72]] and 2 levels of energy intake during the 3 weeks. preceding calving (75% or 125% of estimated requirements) obtained via allowance (m^2^/cow) of fresh pasture composed of mostly perennial ryegrass and white cover [64]. Data in graphs are split between cows receiving Met and those that did not receive Met. Due to the large range and distribution of the metabolomics data variables for liver Met and betaine, they were log transformed. Values reported in the graphs are log values

It is noteworthy that liver Met concentrations were negatively associated with MTR activity (*r* = − 0.35; *P* < 0.001; Fig. 4) and positively with both BHMT (*r* = 0.84; *P* < 0.001; Fig. 4) and CBS (*r* = 0.83; *P* < 0.001; Fig. 4). There was also a positive correlation between liver CBS activity and liver betaine concentrations (*r* = 0.83; *P* < 0.001; Fig. 4), while MTR activity was negatively correlated with betaine concentrations (*r* = − 0.34; *P* < 0.001; Fig. 4). The correlation between CBS and liver Met and betaine suggests that when liver Met synthesis and concentrations are high, flux through the transsulfuration pathway is increased, which fits with published non-ruminant data [30]. Furthermore, the correlation between liver Met and BHMT points to the importance of this enzyme in promoting Met synthesis during the peripartal period. The negative relationship between liver Met with MTR, while weak, could indicate a potential feedback inhibition of Met on MTR activity. Additionally, it is possible that when concentrations of Met increase in the liver, more is used to synthesize choline, which would drive the use of betaine to synthesize Met. This idea is supported by the fact that BHMT was positively correlated with betaine (*r* = 0.84; *P* < 0.001; Fig. 4), which also supports the previous hypothesis that substrate availability is a major regulator of BHMT activity.

In non-ruminants, MAT is regulated by Met availability whereby increased Met leads to greater SAM production [73]. However, in dairy cows, enhanced choline supply (0, 6.25, 12.5 or 25 g/d) during NNB linearly increased hepatic Met, but was associated with a cubic change in hepatic MAT activity; activity was greatest with 12.5 g/d [38]. In sheep, abomasal infusions of Met decreased MAT activity [74]. Thus, in ruminants, beyond a certain threshold the cellular Met concentration may inhibit MAT activity. This hypothesis is further supported by the negative correlation between MTR and MAT activity in the study of Coleman et al. (*r* = − 0.47; *P* = 0.003; Fig. 2), which suggests that greater Met synthesis via MTR would lead to an inhibition of MAT activity. Regarding PEMT, a decrease in SAM level or the ratio of SAM:SAH inhibits transmethylation reactions [75, 76]. Overall, it seems that BHMT may play an important role in synthesizing Met and SAM during the peripartal period. Increasing flux through the transsulfuration pathway via CBS also may be critical for improving production by alleviating oxidative stress.

### Characteristics of one-carbon metabolism pathways in calves

Research on the regulation of enzymes in one-carbon metabolism pathways in pre-ruminants is still ongoing. Classic studies with sheep and more recently with dairy cows have provided evidence for longitudinal adaptations in hepatic activity of these enzymes across various stages of the life cycle [27, 38, 75, 77]. Xue and Snoswell [77] observed that hepatic BHMT activity in lambs increased with age until a ruminant state was reached, whereas MTR activity had a pattern that was opposite to BHMT. In dairy calves, a similar response was observed with BHMT activity increasing between 4 and 14 d of age and decreasing between 28 and 50 d of age [78, 79]. The changes in activity over time in pre-ruminants are likely linked to the establishment of a functional ruminal microbiome, which leads to increased vitamin B_12_ biosynthesis, a cofactor of MTR. Due to ruminal degradation, intestinal bioavailability of choline becomes limited after weaning, which may contribute to the reported decrease in BHMT activity. In rats and humans, CBS activity increases with age [80, 81]. However, in sheep, CBS activity was already almost maximal at 1 d of age compared with 10 and 40 d of age, with a similar trend being reported for hepatic *CBS* mRNA abundance in dairy calves [78]. Although research on calf nutrition has received substantial attention over the past few decades [82], a better understanding of the characteristic associated with one-carbon metabolism could be helpful for adjusting dairy calf nutritional management, consequently achieving better immune function, growth and development during early stages of life.

### Role of dietary methyl donors beyond one-carbon metabolism during the peripartal period

The NNB immediately postpartum is not only associated with fatty liver and a localized inflammatory response, but also impacts immune function in peripheral blood mononuclear cells [83, 84]. Mechanistically, signalling via nutrient-sensing kinases such as the protein kinase B (AKT) and mechanistic target of rapamycin (mTOR), which may modulate metabolism and inflammatory responses, is also altered during this period [85]. Methyl donors can directly and indirectly alter metabolism and the immune system through their crucial roles in signalling pathways, and the synthesis of other functional molecules such as the antioxidants GSH and taurine [5]. Thus, this section of the review will focus on the role that methyl donors play in modulating metabolism, immune function and oxidative stress in dairy cows during the peripartal period (see detailed information in Tables 2, 3, 4, 5).

**Table 2:** One-carbon metabolism genes, proteins, and enzyme activities during late pregnancy and lactation in dairy cows. ↑ = gene/protein abundance and enzyme activity increase; ↓ = gene/protein abundance and enzyme activity decrease

Stage	Dietary manipulation^a^	Tissue/Cells^b^	Effect on abundance of gene and protein, and enzyme activity^c^	Reference
Transition period	RP-Met supply from − 28 d to 30 d relative to calving	Mammary	↑ *GCLC*, *GCLM*, *GSR*, *GPX1*, *ME1*, *FECH*, *FTH1*, *NQO1* gene abundance with RP-Met ↑ *NFE2L2*, *NFKB1*, *MAPK14* gene abundance with RP-Met ↑ NFE2L2 activation with RP-Met	[86]
	RP-Met supply from −28 to 30 d relative to calving	Adipose	↑ *CBS*, *GCLC*, *GSR*, and *GPX1* gene abundance with RP-Met ↑ GPX1, GPX3, GSTM1, and GSTA4 protein abundance with RP-Met Activation of the GSH metabolism	[87]
	RP-Met supply from −28 to 30 d relative to calving	Adipose	↑ AA transporter gene abundance with RP-Met ↑ *AKT1*, *RPS6KB1*, and *EIF4EBP1* gene abundance with RP-Met ↑ Phosphorylated AKT, PPARG and fatty acid synthase protein abundance with RP-Met ↑ mTOR protein abundance (at 30 d in milk) with RP-Met	[88]
	Met supply (RP-Met or Met-analogue) from − 21 to 30 d relative to calving	Liver	↑ *SAHH*, *MAT1A* (at 21 d in milk), *CBS*, *MTR*, and *DNMT3A* gene abundance with Met ↓ *GSS*, *GCLC*, and *SOD1* gene abundance with Met	[27, 89]
	RP-Met or Chol supply from − 21 to 30 d relative to calving	Liver	↓ MTR enzyme activity	[27]
	RP-Met supply from −28 to 30 d relative to calving	Liver	↑ CBS enzyme activity ↑ *MAT1A* gene abundance	[28]
	Pre-partum treatment: 2 BCS categories allowed to 2 levels of energy intake (75% or 125%) in a 2 × 2 factorial design with grazing dairy cows	Liver	↓ MTR and CBS enzyme activity postpartum ↑ BHMT enzyme activity at 7 d in milk ↑ MTR enzyme activity in thin cows ↑ CBS enzyme activity in cows at 125% energy and in thin cows at 125% energy	[64]
	RP-Met or Chol supply from −21 to 30 d relative to calving	PMNL	↓*CBS*, *CTH*, *GSS*, and *GPX1* gene abundance	[90]
	RP-Met supply to prepartum high energy diet from −21 to 30 d relative to calving	PMNL	↓ *GPX1* gene abundance at −10 d from calving in cows receiving RP-Met at high energy diet ↑ *GSR* gene abundance in the post-partum with high energy diet ↑*SAHH* gene abundance in the postpartum with Met supply at high energy compared with low energy	[91]
Mid lactation	*In vitro* Met (40 μmol/L) or Chol (80 mg/dL) supply	PHEP	↑ *MAT1A*, *PEMT*, *SAHH*, *BHMT*, *CSAD*, *GCLC*, and *GSR* abundance with Met ↑*MTR*, *BADH*, *CHDH* abundance with Met or Chol Greatest *CHDH* abundance with Chol ↑ CBS protein abundance with Met	[35]
	*In vitro* Met (Lys:Met ratio of 3.6:1, 2.9:1, or 2.4:1) and Chol (0, 400, or 800 μg/mL) supply	PMNL	↑ *CSAD*, *CTH*, *GSS*, and *GSR* gene abundance with Chol ↑*GSS* and *GSR* gene abundance with Met at Lys:Met ratio of 2.4:1and Chol supply at 400 μg/mL	[19]
	*In vitro* Met (Lys:Met ratio of 3.6:1, 2.9:1, or 2.4:1) with or without LPS challenge	PMNL	↓ *GSR* gene abundance overall with LPS and Met (relevant effect of LPS)	[92]
	*In vitro* Met (Lys:Met ratio of 3.6:1, 2.9:1, or 2.4:1) or Chol (0, 400, or 800 μg/mL) supply under thermoneutral or heat stress conditions	PMNL	↑ mRNA fold-change in abundance of *CBS, CSAD, GSS, GSR, GPX1, TLR2, TLR4, IRAK1,* IL-1β*, IL-10, BAX, BCL2 and HSP70* with Chol ↓ mRNA fold-change abundance of *SAHH* and linear ↑ in *MPO*, *NF-κB*, and *SOD1, CDO1, BAX* and *HSP70* with increasing Met supply	[93]
Cell culture	*In vitro* Met (Lys:Met ratio of 2.9:1, 2.5:1, or 2.0:1)	MAC-T	↑ Intracellular non-essential and essential AA with Met at Lys:Met ratio of 2.0:1 ↑ β-casein and AA transporter gene abundance with Met at Lys:Met ratio of 2.9:1 ↑ mTOR activation with Met at Lys:Met ratio of 2.9:1	[94]
	*In vitro* Met and Arg (Lys:Met 2.9:1 and Lys:Arg 2:1; Lys:Met 2.5:1; Lys:Arg 1:1 or Lys:Met 2.5:1 and Lys:Arg 1:1)	BMEC	↑ AA transporter *SLC7A1* gene abundance with Met at Lys:Met ratio of 2.5:1 ↓ AA transporters gene abundance with Arg at Lys:Arg 1:1	[95]

^a^*RP* rumen-protected, *Met* methionine, *Chol* choline, *Lys* lysine, *Arg* arginine

^b^*PMNL* polymorphonuclear leukocytes cells, *PHEP* primary liver cells enriched with hepatocytes, *MAC-T* immortalized bovine mammary epithelial cell line, *BMEC* primary bovine mammary epithelial cells

^c^*AA* amino acids, *AKT1* AKT serine/threonine kinase 1, *BADH* betaine aldehyde dehydrogenase, *BHMT* betaine homocysteine methyltransferase, *BAX* BCL2 associated X, apoptosis regulator, *CBS* cystathionine β-synthase, *CDO* cysteine dioxygenase, *CHDH* choline dehydrogenase, *CSAD* cysteine sulfinic acid decarboxylase, *CTH* cystathionine-γ-lyase, *DNMT1* DNA (cytosine-5)-methyltransferase 1, *DNMT3A* DNA (cytosine-5)-methyltransferase 3 α, *DNMT3B* DNA (cytosine-5)-methyltransferase 3 β, *EIF4EBP1* eukaryotic translation initiation factor 4E binding protein 1, *FECH* ferrochelatase, *FRAP* Ferric-reducing ability of plasma, *FTH1* ferritin heavy chain 1, *GCLC* glutamate-cysteine ligase catalytic subunit, *GCLM* glutamate-cysteine ligase modifier subunit, *GNMT* glycine N-methyltransferase, *GPX1* glutathione peroxidase 1, *GPX3* glutathione peroxidase 3, *GSR* glutathione reductase, *GSS* glutathione synthase, *GSTA4* glutathione S-transferase Alpha 4, *GSTM1* glutathione *S*-transferase Mu 1, *HSP70* heat shock protein 70, *IL-1β* interleukin 1-β, *IL-6* interleukin 6, *IL-10* interleukin 10, *MAPK14* mitogen-activated protein kinase 14, *MAT* methionine adenosyltransferase, *MAT1A* methionine adenosyltransferase 1A, *ME1* malic enzyme 1, *MPO* myeloperoxidase, *mTOR* mechanistic target of rapamycin, *MTR* 5-methyltetrahyrdofolate-homocysteine methyltransferase, *NFE2L2* nuclear factor erythroid 2-like 2, *NFKB1* nuclear factor κβ subunit 1, *NQO1* NAD(P)H quinone dehydrogenase 1, *ORAC* oxygen radical absorbance capacity, *PEMT* phosphatidylethanolamine methyltransferase, *PON* paraoxanase, *PPARG* peroxisome proliferator activated receptor gamma, *ROM* reactive oxygen metabolites, *RPS6KB1* ribosomal protein S6 kinase B1, *SAA* serum amyloid A, *SAHH* *S*-adenosylhomocysteine hydrolase, *SOD1* superoxide dismutase 1

**Table 3:** One-carbon metabolism genes, proteins, and enzyme activities in early age of dairy calves and uterine environment. ↑ = gene/protein abundance and enzyme activity increase; ↓ = gene/protein abundance and enzyme activity decrease

Stage	Dietary manipulation^a^	Tissue/Cells^b^	Effect on abundance of gene and protein, and enzyme activity^c^	Reference
Calves^d^	Maternal RP-Met supply for 21 d before calving	Liver	↑ *BHMT*, *SAHH*, and *CBS* gene abundance at 4 and 14 d of age in Met calves ↑ *MAT1A* in Met calves, overall ↑ GCLC and GSR at 4 d of age in Met calves ↑ *BHMT*, *SAHH*, *DNMT1*, *DNMT3A*, *DNMT3B*, *CSAD*, *CBS*, *GCLC*, *GSR* with aging until 50 d DNA methylation might be an important component of the physiologic adaptations of calf liver ↑ BHMT enzyme activity with aging until 28 d of age	[78]
	Maternal RP-Met supply for 28 d before calving	Liver	↑ BHMT enzyme activity Met calves at 14 d of age ↓ CBS enzyme activity in Met calves, increasing at 14 and 28 d of age ↓ MTR enzyme activity in Met calves at 4 and 50 d of age ↑ *MTR*, *DNMT3A*, and *BADH* gene abundance in Met calves	[79]
	Maternal RP-Met supply for 21 d before calving	PMNL	↑ *CBS* and *CTH* gene abundance at birth ↓ *GSR* gene abundance at birth ↑ *CBS*, *GCLC*, *GSS*, and *GPX1* with aging	[96]
	Maternal RP-Met supply for 28 d before calving	PMNL	↑ *GPX1* gene abundance in Met calves	[97]
Uterine	Maternal RP-Met supply for 28 d before calving	Placenta	↑ AA and glucose transporter gene abundance with RP-Met ↑ *MTOR* and *RPS6KB1* gene abundance with RP-Met ↑ mTOR activation with RP-Met	[8]
	Maternal RP-Met supply for 28 d before calving	Placenta	↑ TCA cycle and transsulfuration intermediates in Male calves with RP-Met ↑ MTR activity in Male calves with RP-Met ↑ One-carbon metabolism intermediates in Female calves with RP-Met ↑ *DNMT3A* gene abundance in Female calves with RP-Met ↓ Global DNA methylation in Female calves with RP-Met	[98]
	Maternal RP-Met supply from calving until embryo flushing (around 70 d postpartum)	Embryos	↓ Embryonic development genes expression (*VIM*, *IFI6*, *BCL2A1*, *TBX15*) with RP-Met ↓ Immune response genes expression (*NKG7*, *TYROBP*, *SLAMF7*, *LCP1*, *BLA-DQB*) with RP-Met	[12]

^a^*RP* rumen-protected, *Met* methionine

^b^*PMNL* polymorphonuclear leukocytes cells

^c^*AA* amino acids, *BADH* betaine aldehyde dehydrogenase, *BCL2A1* BCL2 related protein A1, *BHMT* betaine homocysteine methyltransferase, *BLA-DQB* MHC class II antigen, *CBS* cystathionine β-synthase, *CSAD* cysteine sulfinic acid decarboxylase, *CTH* cystathionine-γ-lyase, *DNMT1* DNA (cytosine-5)-methyltransferase 1, *DNMT3A* DNA (cytosine-5)-methyltransferase 3α, *DNMT3A* DNA methyltransferase 3A, *DNMT3B* DNA (cytosine-5)-methyltransferase 3β, *GCLC* glutamate-cysteine ligase catalytic subunit, *GPX1* glutathione peroxidase 1, *GSR* glutathione reductase, *GSS* glutathione synthase, *IFI6* interferon alpha inducible protein 6, *LCP1* lymphocyte cytosolic protein 1, *MAT1A* methionine adenosyltransferase 1A, *MTOR* mechanistic target of rapamycin, *MTR* 5-methyltetrahyrdofolate-homocysteine methyltransferase, *RPS6KB1* ribosomal protein S6 kinase B1, *SAHH* *S*-adenosylhomocysteine hydrolase, *SLAMF7* signaling lymphocyte-activating molecule family member 7, *TBX15* T-box transcription factor15, *TCA* tricarboxylic acid, *TYROBP* transmembrane immune signaling adaptor TYROBP, *VIM* vimentin

^d^Calves were evaluated from birth to 50 d of age

**Table 4:** Biomarkers of inflammatory response, liver function and oxidative status in dairy cows supplemented with methyl donors according to stage of lactation. ↑ = biomarker increase concentration; ↓ biomarker decrease concentration

Stage	Dietary manipulation^a^	Effect on biomarker concentrations^b^	Reference
Transition period	RP-Met or RP-Chol supply from −21 to 30 d relative to calving	↓ IL-1β and haptoglobin with RP-Met ↑ IL-6 with RP-Met ↑ PON, albumin and cholesterol with RP-Met ↑ Liver GSH and GSR with RP-Met	[25]
	RP-Met supply from − 28 to 30 d relative to calving	↓ Haptoglobin with RP-Met ↓ ROM with RP-Met ↑ IL-6 with RP-Met ↑ PON, albumin and cholesterol with RP-Met ↑ FRAP, β-carotene and tocopherol with RP-Met ↑ Liver GSH and GSR with RP-Met	[29]
	RP-Met or RP-Chol supply from − 21 to 21 d relative to calving	↑ IL-2 with RP-Met and RP-Chol ↓ IL-6 and TNF with RP-Met and RP-Chol ↑Total antioxidant capacity, GPX and vitamin E with RP-Met and RP-Chol ↓ Total bilirubin, ALP and MDA with RP-Met and RP-Chol ↑ TPP with RP-Chol ↓ GPT and GOT with RP-Chol ↓ BUN with RP-Met	[99]
	Met supply (RP-Met or Met-analogue) from − 21 to 30 d relative to calving	↓Ceruloplasmin and SAA with Met ↑ ORAC with Met	[33]
	RP-Met or RP-Chol supply from − 21 to 30 d relative to calving	↓ IL-1β after whole blood LPS challenge postpartum with RP-Met ↑ Neutrophil and monocyte phagocytosis and oxidative burst postpartum with RP-Met ↑ Monocyte phagocytosis with RP-Chol	[100]
	RP-Chol at 25 g/d prepartum and 50 g/d postpartum of Chol ions from − 21 to 60 d relative to calving	↓ Liver TLI and TAG with Chol ↓ Liver TAG:glycogen ratio with Chol ↑ Plasma TAG with Chol	[101]
Mid lactation	Chol ions at 0, 6.25, 12.5 or 25 g/d through abomasal infusion with restricted intake management during 4 d	↓ Liver CBS activity linearly with Chol ↑ Liver taurine with Chol ↑ Plasma α-tocopherol and β-carotene with Chol ↓ Plasma AST and bilirubin with Chol ↑ PON with Chol	[37]
Dry off	RP-Chol at 0, 6.5, 12.9, 19.4 or 25.8 g/d of Chol ions during 15 d with feed-restriction and fat-loading management	↑ Liver lipotropic effects with up to 25.8 g/d of Chol ions in NNB cows ↑ Liver glycogen and TAG with 6.5 g/d of Chol ions in NNB cows	[102]

^a^*RP* rumen-protected, *Met* methionine, *Chol* choline

^b^*ALP* alkaline phosphatase, *AST* aspartate aminotransferase, *BUN* blood urea nitrogen, *CBS* cystathionine β-synthase, *FRAP* Ferric-reducing ability of plasma, *GOT* glutamic oxalacetic transaminase, *GPT* glutamate pyruvate transaminase, *GPX* glutathione peroxidase, *GSH* glutathione, *GSR* glutathione redutase, *IL-1β* interleukin-1β, *IL-2* interleukin-2, *IL-6* interleukin-6, *LPS* lipopolysaccharide, *MDA* malondialdehyde, *NNB* negative nutrient balance, *ORAC* oxygen radical absorbance capacity, *PON* paraoxanase, *ROM* reactive oxygen metabolites, *SAA* serum amyloid A, *TAG* triacylglycerol, *TLI* total lipid, *TNF* tumor necrosis factor-α, *TPP* total plasmatic protein

**Table 5:** Main effects of experiments including betaine, vitamin B_12_ and folic acid supplementation in dairy cows according to stage of lactation. ↑ = increase effect; ↓ decrease effect

Stage	Dietary manipulation^a^	Main effects^b^	Reference
Transition period	CON or Bet from close-up until 8 weeks postpartum vs. CON or Bet from dry off until 8 weeks postpartum	↑ Milk yield and milk fat in Bet cows enrolled at dry off ↑ NEFA and BHBA postpartum Bet in cows enrolled at dry off	[103]
	CON or RP-Bet at 20 g/d from 4 weeks prepartum until 6 weeks postpartum	↑ Feed efficiency (ECM/DMI) with RP-Bet ↑ BW loos postpartum with RP-Bet ↑ BHB at d 7 postpartum with RP-Bet ↑ TPP and plasma globulin with RP-Bet ↓ Plasma glucose with RP-Bet	[104]
	FA at 0, 120 or 240 mg/500 kg of BW from 1 week prepartum to 1 week postpartum	↑ Milk yield with FA 120 mg/500 kg of BW	[105]
	FA at 0 or 2.6 g/d and B_12_ at 0 or 0.5 g/d in 2 × 2 factorial design from 3 weeks prepartum to 8 weeks postpartum	↑ Milk yield and milk protein with FA ↓ Milk urea N with B_12_ ↑ Liver phospholipids with B_12_	[106]
	Basal diet with restricted Met or basal diet + RP-Met. In each group half received weekly vitamin injections of FA 160 mg + B_12_ 10 mg	↑ Milk and plasma level of FA and B_12_ with vitamin ↑ Milk yield with vitamin ↑ Milk lactose, milk protein and total solids with vitamin	[107]
Mid lactation	Bet at 0, 50, 100 or 150 g/d during 30 d	↑ Milk yield and milk fat linearrly with Bet	[108]
	Bet at 0, 25, 50, or 100 g/d during 16 d	↑ Milk yield with Bet 100 g/d ↓ Milk protein in Bet cows compared with CON	[109]
	CON, 20 g/d of RP-Met, 45 g/d RP-Bet, or 40 g/d of RP-Chol in limited Met diet during 70 d	↑ Milk yield in RP-Chol ↑ Milk protein in RP-Chol vs. CON or RP-Bet ↑ Milk fat in RP-Chol vs. RP-Met	[110]
	Bet at 0, 10, 15 or 20 g/d for 8 weeks during heat stress period	↑ DMI with Bet ↑ Milk yield, milk lactose and milk protein with Bet ↑ Plasma cortisol, GPX, SOD and MDA with Bet ↓ Plasma HSP70 with Bet	[111]
	Bet at 0, 57 or 114 mg/kg of BW for 2 weeks in thermoneutral and 2 weeks in heat stress conditions	↑ Milk yield with Bet 114 mg/kg of BW in thermoneutral conditions ↓ Respiration rate with Bet in heat stress ↑ Rectal temperature with Bet in heat stress ↑ Plasma glucose with Bet 114 mg/kg of BW in heat stress	[112]
	Basal diet with RP-Met and FA with or without weekly B_12_ 10 mg injections from 4 to 18 weeks of lactation	↑ ECM, milk yield of solids, fat and lactose with B_12_ ↑ Blood hemoglobin and serum vitamin B_12_ with B_12_	[113]
	RP-FA at 0 or 118 mg/d during 90 d	↑ Milk yield, FCM and milk protein with RP-FA ↑ Plasma albumin with RP-FA	[114]
Whole lactation	FA at 0, 2, or 4 mg/kg of BW from 4 weeks prepartum to 305 d of lactation	↑ Serum and milk folate level with FA ↑ Multiparous milk yield with FA	[115]

^a^*RP* rumen-protected, *Bet* Betaine, *Met* methionine, *Chol* choline, *FA* folic acid, *B*_*12*_ vitamin B_12_, *BW* body weight, *CON* control without supplement

^b^*BHBA* β-hydroxybutyrate, *DMI* dry matter intake, *ECM* energy-corrected milk, *FCM* fatty-corrected milk, *GPX* glutathione peroxidase, *HSP70* heat shock protein 70, *MDA* malondialdehyde, *NEFA* non-esterified fatty acids, *SOD* superoxide dismutase, *TPP* total plasmatic protein

### Methionine and production performance

In terms of production, a meta-analysis using 64 papers concluded that post-ruminal Met supply contributes to increased milk yields and milk protein and fat [116]. During the peripartal period specifically, studies have consistently reported an increase in milk, protein and fat yields with RPM [10, 11, 25]. Supplemental Met has also had a positive response in maintaining consistent rates of DMI prepartum and faster and greater rates of DMI during the first 30 to 60 d postpartum [10, 11, 25]. The mechanisms behind the DMI effect are still unclear; however, the increases in milk protein yield in response to RPM supply might be associated with activation of mTOR, a serine/threonine kinase that plays a central role in regulating protein synthesis and cell growth [117].

### Methionine and mammary tissue

In vitro studies with bovine mammary epithelial cells have demonstrated that supplying Met upregulates abundance of AA transporters, increasing flux into the cell and promoting activation of mTOR to stimulate protein synthesis [94, 95, 118–120]. From a mechanistic standpoint, data from non-ruminants indicate that SAM generated from Met can indirectly activate mTOR. Upon synthesis, SAM can bind to *S*-adenosylmethionine sensor upstream of mTOR (SAMTOR), a protein that inhibits mTOR complex 1 (mTORC1) by interacting with gap activity toward rags 1 [121]. When SAM binds to SAMTOR, it inhibits the association of SAMTOR and gap activity toward rags 1, allowing mTORC1 to be activated [121]. This mechanism has not been studied in dairy cows; however, we speculate that it is another potential route by which Met activates mTOR in dairy cattle. Overall, available data suggest that improved milk protein yield with RPM may be due primarily to enhanced flux of AA and protein synthesis in the mammary gland. More *in vivo* studies are needed to have a better understanding of the role of Met in regulating milk protein synthesis.

Another important modulatory role in assessing the benefits of Met supplementation in mammary tissue was observed through its antioxidant effects. It is well-known that mammary epithelial cells in high-yielding cows are prone to oxidative stress, which can cause significant damage during lactation [122]. The nuclear factor erythroid 2-like 2 (NFE2L2) plays an important role in controlling oxidative damage through regulating a wide range of antioxidant genes [123]. Enhanced RPM supply during the periparturient period increased abundance of phosphorylated NFE2L2 in mammary tissue, leading to upregulated mRNA abundance of *GPX1*, *GCLC,* glutamate cysteine ligase modifier subunit, malic enzyme 1, ferrochelatase and ferritin heavy chain 1 (genes involved in GSH and iron metabolism), suggesting that Met supply might alleviate oxidative stress via activation of the NFE2L2 pathway [86].

### Methionine and subcutaneous adipose tissue

Human and rodent studies have demonstrated that branched-chain AA are crucial regulators of the mTOR pathway [124]. Of note, a recent study reported that compared with liver and skeletal muscle, SAT had the greatest mRNA abundance of enzymes associated with branched chain AA catabolism in peripartal cows [125]. Thus, due to inherent differences in AA metabolism among tissues, it is likely that Met might play different roles across tissues during the transition period. Beyond the mammary gland, enhanced supply of Met from − 28 to 60 d relative to parturition upregulated both mRNA and protein abundance of some AA transporters and phosphorylated (p) mTOR and p-AKT in dairy cow subcutaneous adipose tissue (SAT), suggesting it might promote insulin sensitivity [88].

Additionally, the benefits of RPM supplementation on antioxidant metabolism have also been detected in SAT. Evaluating if RPM could increase the abundance of genes and proteins related to GSH and NFE2L2 metabolism in SAT, our previous work revealeded that cows receiving RPM from − 28 d to 30 d relative to calving had increased mRNA (e.g. *CBS*, *GCLM*, *GSR*) and protein (e.g. GPX1, GPX3, GSTM1, GSTA4) abundance of enzymes related to GSH metabolism in SAT [87]. As demonstrated in mammary tissue data, our results in SAT suggest that exogenous Met might be a potential modulator of the NFE2L2 pathway by altering GSH synthesis, which will contribute to decreased oxidative overload that these cows face during the transition period. However, due to the greater DMI with RPM in this study, we should take this conclusion with caution. Additionally, murine studies reported lower mRNA and protein abundance and lower activity of cystathionine γ-lyase (one of the key enzymes associated with the transsulfuration pathway) in SAT [81, 126]. Thus, *in vitro* studies using bovine adipocytes stimulated with free radicals such as hydrogen peroxide are warranted to investigate the role of Met in mediating the NFE2L2 pathway and GSH synthesis.

### Methionine and immune cells

Enhanced post-ruminal supply of Met during the peripartal period is associated with enhanced immune cell function. Supplying RPM from 21 d prepartum until 30 d postpartum increased whole blood neutrophil phagocytosis [10]. Furthermore, RPM supplementation during the peripartal period enhanced *in vitro* neutrophil phagocytosis capacity and oxidative burst activity [25, 29]. In one study, supplemental Met was associated with lower abundance of genes related to inflammation (e.g. *IL1B*, *TLR2*, *NFKB1* and *STAT3*) and oxidative stress (e.g. *CBS*, *GPX1*, *GSS*, and *SOD2*) in isolated PMNL as well as an increase in plasma taurine, suggesting a better redox and reduced inflammatory status [90]. In addition, those cows were used for an ex vivo whole blood challenge with lipopolysaccharide (LPS) and a hyper-response in interleukin-1β was observed around parturition [100]. However, RPM supplementation dampened this hyper-response, likely through reductions in oxidative stress [100].

Recent work also investigated the effects of incubating bovine blood PMNL with Met and/or choline and observed that supplemental Met coupled with adequate choline enhanced mRNA abundance of Toll-like receptor (*TLR*) 2 and L-selectin, which are involved in pathogen recognition and cell-adhesion mechanisms, respectively [19]. Cells incubated without choline had greater mRNA abundance of interleukins and genes involved in GSH metabolism; this effect, however, was ameliorated by supplementing additional Met [19]. More recently, Lopreiato et al. [93] used isolated PMNL for incubations with 3 ratios of Lys:Met (3.6:1, 2.9:1 or 2.4:1) under thermoneutral or heat stress conditions. They reported a decrease in mRNA abundance of S-adenosylhomocysteine hydrolase and linear increases in myeloperoxidase, *NFKB1*, and *SOD1* with increasing Met supply. In addition, increasing supply of Met during heat stress also upregulated mRNA abundance of cysteine dioxygenase 1 and BCL2 associated X apoptosis regulator and heat shock protein 70 suggesting an improvement in antioxidant and cytoprotective characteristics [93]. Hence, transcriptional changes in bovine immune cells may at least partly explain the reduced inflammation status and improved immune function in peripartal cows fed RPM.

Concerning the cytoprotective mechanisms against oxidative stress through the use of GSH, PMNL supplemented with Met *in vitro* are characterized by a greater abundance of *GPX1*, encoding glutathione peroxidase-1, which reduces hydrogen peroxide to water by using GSH as co-factor [19]. These results confirmed not only the critical role of Met in the GSH antioxidant pathway, but that Met plays a key role in protecting immune cells because GSH is the most-potent cellular antioxidant agent in PMNL cells during an inflammatory response [127].

A decrease in plasma interleukin-1β and haptoglobin concentrations coupled with an increase in albumin in response to feeding RPM suggest an alleviation of inflammation by Met in peripartal dairy cows [25, 29, 89, 99]. Besides aspects of the immune and antioxidant systems, *in vivo* studies have consistently detected improvements in plasma biomarkers of liver function such as increases in paraoxonase activity and cholesterol concentrations with RPM [25, 29, 33], which is likely linked to the reduction in inflammation and oxidative stress. Thus, the consistent changes across studies in plasma biomarker concentrations, mRNA and protein abundance indicate that enhanced Met supply during the peripartal period contributes to reduced oxidative stress and inflammation status. However, the exact mechanisms need to be studied further.

### Choline and production performance

Two recent meta-analyses have been performed to investigate better the effects of RPC supplementation on milk production, metabolic health and postpartal disorders [128, 129]. Although in one of the meta-analysis there was no effect of RPC on DMI during the prepartal period, postpartal DMI increased in both analyses [128, 129]. The increase in DMI postpartum in both analyses was associated with increases in milk yield [128, 129]. While there was no effect of RPC on milk fat or protein content, the increase in milk yield with RPC drove an increase in yields of both [128]. It is noteworthy that this meta-analysis revealed no dose-dependent effects of RPC on DMI or milk production [128], which contrasts recent work by Coleman et al. [38] where abomasal infusions of 0, 6.25, 12.5 and 25 g/d choline ion during a feed restriction-induced NNB increased milk yield linearly. In the same way, the meta-analysis by Arshad et al. [129] revealed that choline ion supplementation during the transition period promoted a linear increase in milk yield, energy-corrected milk, fat and protein. The analysis also revealed an interaction between choline and metabolizable Met during the postpartum period, i.e. as total Met percentage in the metabolizable protein increased, the positive response to RPC on milk production, energy-corrected milk, and milk protein decreased. Taken together, we speculate that the differences between the study by Coleman et al. [38] and the results of the meta-analyses by Humer et al. [128] and Arshad et al. [129] could be due to differences between studies in terms of rumen-protected products used, diet formulation and the supply of other methyl donors.

### Choline and triacylglycerol accumulation in the liver

In dairy cattle, much of the *in vivo* work has focused on the use of RPC supplementation during the peripartal period to enhance PC and VLDL synthesis in order to limit the development of fatty liver. Changes in hepatic TAG with RPC have been inconsistent. However, several studies have reported a reduction in liver TAG with RPC [101, 130, 131] and enhanced post-ruminal choline supply during NNB [38]. Although the meta-analysis by Humer et al. [128] did not assess liver TAG concentrations, the authors discussed the inconsistency in the response pattern reported in the literature. The high variation on the average postpartal TAG concentration, including cows with extremely high TAG concentrations, was highlighted as a reason that “hides” the potential effects of RPC supplementation. This factor could only be disregarded if the analysis is performed with a greater number of cows. A recent study evaluating the effects of RPC on immunometabolic status of peripartal cows revealed greater postpartal hepatic and plasma TAG concentrations in RPC-fed cows, which authors suggested were associated with increased milk yield in the absence of increased DMI [132]. The meta-analysis conducted by Arshad et al. [129] demonstrated no differences in liver tissue TAG concentrations during the postpartum period between cows with or without RPC supplementation. According to the authors, most of the positive effects of RPC on TAG concentrations were evident in dry cows [102] and, thus, in the postpartum period when challenged by the high demand for nutrients for milk synthesis, the positive effects of RPC can be diluted.

In those studies that demonstrated positive effects of RPC supply on liver TAG concentrations, the reduction was likely supported by increased PC synthesis via the CDP-choline pathway, with mRNA abundance of enzymes in that pathway being increased both *in vitro* [27] and *in vivo* with enhanced choline supply [35]. Enhanced choline supply with RPC or abomasal infusions also has been associated with increased mRNA abundance of apolipoproteins such as apolipoprotein A5, and apolipoprotein B100 [38, 133], which are required for VLDL synthesis in the liver [134]. Together, those changes indicate that enhanced VLDL synthesis might be a mechanism behind reduced liver TAG content with choline.

Coleman et al. [38] reported an increase in the mRNA abundance of carnitine palmitoyl transferase 1A (a gene involved in fatty acid oxidation) and solute carrier family 22 member A5 (*SLC22A5*; a carnitine transporter) with enhanced abomasal choline supply during NNB; furthermore, decreased CBS activity along with increased MTR and BHMT activity in the liver were observed in response to abomasal choline supply. These changes were also associated with an increase in hepatic carnitine concentrations [37]. Thus, it is possible that post-ruminal choline supply during NNB reduces entry of homocysteine to the transsulfuration pathway, potentially supporting remethylation to Met by acquiring a methyl group from betaine. Methionine could then be metabolized to SAM as described earlier. Subsequently, SAM can be used to produce trimethyl lysine, consequently leading to greater carnitine synthesis [76].

Goselink et al. [133] observed an increase in *SLC22A5* and fatty acid transport protein 5 with RPC supplementation for 3 weeks pre- and 6 weeks post-calving. Together with Coleman et al. [38], such changes suggest that supplying choline may also lead to reductions in hepatic TAG through increases in fatty acid oxidation. These genes have been indicated as targets of the transcription factor peroxisomal proliferator activated receptor α (PPARA). While neither study observed differences in *PPARA* [38, 133], *SLC22A5* and *CPT1A* are targets of PPARA in ruminants [135]. Thus, increases in *SLC22A5* and *CPT1A* suggested that an increase in PPARA activity with choline was the mechanism behind improved fatty acid oxidation. Overall, enhanced choline supply may reduce liver TAG through a combination of both increased VLDL synthesis and increased fatty acid oxidation.

### Choline and immunometabolic status

Choline has been observed to alleviate oxidative stress and inflammation in non-ruminants [136–138], which can help improve liver function as well. While most studies on choline in dairy cattle have not focused on this aspect of choline metabolism, several studies have reported potential benefits of choline on oxidative stress, inflammation and liver function in dairy cattle. In the study by Zhou et al. [25], RPC supplementation during the peripartal period did not alter plasma biomarkers of oxidative stress and inflammation. However, choline increased monocyte phagocytosis capacity, suggesting a potential improvement in monocyte function [100]. In a study by Zenobi et al. [139], supplementing RPC at 12.9 g/d from 21 d prepartum through 21 d postpartum decreased TNFα protein production, mRNA abundance of *IL1B*, *TNF* and C-X-C motif chemokine ligand 8 and CD80 in blood leukocytes that were stimulated with LPS regardless of whether energy was fed in excess or at maintenance during the dry period. Furthermore, at 17 d postpartum the proportions of phagocytic neutrophils and those undergoing oxidative burst increased with RPC regardless of energy intake level [139]. Using a feed restriction model, Zenobi et al. [102] observed no differences in plasma haptoglobin, a marker of inflammation, when increasing amounts of choline ion were fed as RPC (0, 6.5, 12.9, 19.4 or 25.8 g/d). Similarly, Coleman et al. [37] also did not report differences in biomarkers of inflammation with choline supply during a feed restriction-induced NNB. However, enhanced choline supply during NNB did increase plasma concentrations of α-tocopherol and β-carotene, suggesting an improvement in antioxidant status [37]. Furthermore, choline supply during NNB tended to increase hepatic taurine concentrations in those cows, suggesting a potential improvement in liver antioxidant status [37]. When RPC was supplemented for 21 d pre- and postpartum, Sun et al. [99] observed an improvement in antioxidant responses via increased total antioxidant capacity, GPX and vitamin E and decreased malondialdehyde. They also observed an improvement in immune function and reduction in inflammation, with an increase in IL-2 and the ratio of CD4^+^/CD8^+^ cells in blood, and a decrease in circulating IL-6 and TNF-α [99]. While improvements in antioxidant status likely stem from improved taurine and GSH synthesis with enhanced choline supply, the increase in circulating vitamin E is potentially linked to an improvement in VLDL synthesis since VLDL is known to accompany the incorporation and transport of vitamin E [140]. Enhanced immune cell function is potentially linked to mTOR signalling, and choline has been observed to modulate immune function via mTOR in organs such as spleen and head kidney in fish [141]. However, this mechanism needs to be verified in dairy cattle and should be considered in future studies.

Choline may play an important role in modulating immune function during periods of heat stress. Lopreiato et al. [93] isolated PMNL from cows (153 DIM) and incubated them with 0, 400 or 800 μg/mL of choline under thermoneutral or heat stress conditions. They observed that mRNA abundance of *CBS*, *GSS*, *GSR, GPX1, TLR2, TLR4*, *IL1B*, *IL10,* heat shock protein 70, BCL2 associated X increased with choline supply. These results again highlight the potential role of choline in mediating antioxidant, cytoprotective and immune mechanisms in PMNL, specifically during periods of heat stress.

Changes in biomarkers of liver function with choline supplementation have been inconsistent across studies in dairy cows. Cholesterol has been one of the most-widely measured markers of liver function, as increased concentrations would be associated with increased lipoprotein levels in the blood [142]. However, changes in cholesterol in response to RPC supply in peripartal cows have been inconsistent, with some reporting no changes [25, 101, 143–145], others an increase [102, 146] and one study a decrease [99]. The inconsistency in cholesterol in response to RPC is likely related to the variation in the degree of change in hepatic TAG across studies, as described earlier. Zhou et al. [25] reported no differences in bilirubin, aspartate aminotransferase, γ-glutamyl transferase or paraoxonase with RPC in periparturient cows. Elek et al. [101] also reported no differences in aspartate aminotransferase when they supplemented 25 g/d choline chloride for 21 d prepartum and 50 g/d choline chloride for 60 d postpartum. The same result was observed by Zahra et al. [143] when RPC was fed for 21 d pre- and 28 d postpartum. However, Sun et al. [99] observed decreases in bilirubin, alkaline phosphatase and γ-glutamyl transferase when RPC was fed during the peripartal period, suggesting that RPC cows had a better or unimpaired liver function than the controls (without RPC supply). More recently, cows receiving abomasal infusions of choline during a feed restriction-induced NNB had greater paraoxonase and lower aspartate aminotransferase and bilirubin than cows not receiving choline, suggesting better liver function in cows receiving abomasal choline compared with controls [37]. Overall, studies thus far suggest a beneficial effect of enhanced choline supply during periods of NNB in dairy cattle on antioxidant and immune responses and liver function, which may support improved milk production. However, more work is needed to fully understand the mechanisms behind those changes.

### Betaine

Betaine plays an important role in the remethylation of homocysteine to Met via BHMT. By promoting the production of Met, betaine has the potential to promote synthesis of PC, GSH, taurine and SAM. Additionally, betaine is an osmolyte [147], which gives it a potential role in pulling water to the mammary gland to help increase milk yields. In dairy cows, there has been limited research on the effects of betaine, with studies primarily focusing on production parameters. Wang et al. [108] fed 0, 10, 50, and 100 g/d of anhydrous betaine for 30 d to mid-lactation cows and observed linear increases in milk and fat-corrected milk yields, and a quadratic increase in milk fat. Similarly, supplementation of 100 g/d of rumen-unprotected betaine for 16 d increased milk and milk protein yields in mid-lactation Holstein dairy cows [109]. However, in another study, milk yields were not altered when rumen-protected betaine was supplemented from 28 to 91 DIM [110]. There was also no difference in plasma concentrations of VLDL or cholesterol, suggesting that betaine did not alter export of liver TAG [110]. It should be noted, however, that the basal diet in that study was limiting in Met [110], which could explain the lack of differences. In peripartal cows, supplementation of a betaine-containing liquid supplement for 60 d prepartum and 56 d postpartum increased milk yields and fat-corrected milk [103]. Interestingly, plasma concentrations of Met were not altered by betaine supply in this study [103], suggesting that remethylation of homocysteine to Met via BHMT was not enhanced. Additionally, when rumen-protected betaine was supplemented for 4 weeks prepartum and 6 weeks postpartum, milk yields and composition were not altered, but rumen-protected betaine tended to increase feed efficiency compared with controls [104].

There are two studies investigating the effects of betaine during heat stress, where NNB also occurs due to a drop in DMI. In the first study, cows experiencing heat stress were fed 0, 10, 15 or 20 g/d betaine from rumen-protected betaine for 8 weeks, with 15 g/d betaine leading to increased DMI, milk yield and milk protein compared with 0 g/d [111]. They also observed a potential improvement in antioxidant responses, with an overall increase in plasma GPX, SOD, and total antioxidant capacity [111]. In the second study, cows were fed 0, 57 or 114 mg betaine/kg of body weight (BW) for 7 d at thermoneutral conditions followed by 7 d of heat stress [112]. During thermoneutral conditions, cows with the highest betaine intake had significantly greater milk yields than controls, but there was no difference during heat stress [112]. The difference between studies could be due to the use of rumen-protected betaine vs. pure betaine in the second study. Overall, supplemental betaine during periods of NNB may have beneficial effects on production as well as oxidative stress. However, more work is needed, particularly with rumen-protected sources, to understand these potential effects and the mechanisms behind them.

### Folic acid

In addition to its role in one-carbon metabolism, folic acid (FA) regulates the synthesis and stabilization of DNA and RNA and plays an important role in immune function [148]. Under normal conditions, ruminal microorganisms produce enough folate for ruminants [3]. However, to maintain high milk yields during the peripartal period supplemental folate may be needed to meet requirements [113]. Thus, supplemental folate may have a role in helping to promote immune function and metabolism during periods of NNB. In one of the first studies with FA in dairy cows, cows injected with FA from 45 d after mating to 6 weeks postpartum had increased milk production and milk protein percent during the last half of the lactation curve; however, there were no differences during early lactation [149]. Girard and Matte [115] also supplemented 0, 2 or 4 mg FA/kg of BW for 4 weeks prepartum through 305 d lactation and reported a parity effect on milk production; there was little effect of FA on primiparous cows, but multiparous cows receiving FA had increased milk yields. Recently, Khan et al. [105] supplemented 0, 120 or 240 mg FA/500 kg BW 1 week pre- and postpartum and followed through 4 months of lactation. Over the first 3 months of lactation cows supplemented with the middle dose of FA had greater milk yields, but there were no differences in milk fat or protein [105]. Lymphocytes from those cows were also used for transcriptomic analysis, which revealed altered immune signalling with the 120 mg dose improving immune-related genes and inflammatory pathways [150], as well as metabolic pathways (e.g. glycolysis, GSH metabolism and folate biosynthesis) [105]. In mid-lactation cows, Du et al. [114] observed an increase in milk, FCM and milk protein yields when rumen-protected folate was supplemented for 90 d. They also observed an increase in plasma albumin with FA, suggesting a potential reduction in inflammation.  However, no other parameters of inflammation were measured [114].

Other available studies on FA in dairy cows have focused on the supply of FA in combination with vitamin B_12_ delivered through intramuscular injections, or in combination with Met [106, 107, 151–157]. The basis for such studies is due to the need for vitamin B_12_ by MTR (Fig. 1), and the fact that Met supply, as described earlier, may also modulate MTR activity. Those studies suggest that FA may have a beneficial effect on milk production and improve energy partitioning, but those effects may be dependent on vitamin B_12_ and Met. However, more work is needed, particularly with feeding FA and vitamin B_12_ since the use of weekly intramuscular injections limits the feasibility of application on farm. Overall, there seems to be a benefit of enhanced FA supply on production and metabolism during periods of NNB such as the peripartal period; however, a further studies are needed to fully understand the mechanisms behind changes in production and metabolism.

Overall, there is a clear benefit to methyl donor supplementation during the peripartal period. These effects stem from changes beyond one-carbon metabolism, with improvements being observed in production, inflammation, immune function and liver function. While most work thus far has focused on Met and choline supply, there is growing evidence that FA and vitamin B_12_ may play important roles during the peripartal period. However, more work is needed to fully understand the mechanisms by which all of these methyl donors alter immunometabolism of dairy cows.

### Implications of maternal methyl donor supply on calf growth and immunometabolic status

#### Methionine

The peripartal period is characterized by dramatic fetal growth, and maternal nutritional status can affect nutrient availability by the fetus subsequently changing their physiology and metabolism [158]. For instance, in ruminants, it is documented that maternal energy level during late-gestation alters growth and immunometabolic status of their offspring [159–161]. Epigenetic changes such as DNA methylation and histone modifications [162] during this period may influence fetal and placental developmental [163]. There is evidence that manipulating maternal dietary supply of methyl donors may induce epigenetic changes in the fetus, which can alter growth and development in the postnatal period [164]. In dairy cattle, supplementing Met during the preimplantation period (1 through ~ 70 d postpartum) had a clear effect on gene transcription of the developing embryo [12]. Thus, while the main focus of this review is on the effects of methyl donors during the peripartal period, it is worth noting that supplementing methyl donors prior to implantation may also have metabolic and immune benefits on fetal programming.

Recently, our group reported that nutrient transporters and signaling proteins in term placenta are altered by maternal Met supply during late gestation. Specifically, when cows were supplemented with RPM during the last 28 d of gestation the mRNA abundance of AA and glucose transporters, as well as abundance of proteins involved in mTOR signaling were upregulated in placenta [8]. It is, however, important to note that cows supplemented with RPM had greater DMI pre-calving [11]. Thus, this may have contributed to those changes, as more nutrients would have been available with greater intakes. Interestingly, there were also sex divergent effects in the concentrations of metabolites, where placenta from female calves born from dams receiving RPM had greater concentrations of Met and SAM, but there was no differences in male calf placenta [98]. Corresponding to this increase in SAM, the abundance of *DNMT3A* and *DNMT3B* was upregulated as well in female placenta with maternal RPM. However, despite those effects, global DNA methylation was lower in placenta from female RPM calves compared with control calves [98].

These changes in transporters and epigenetic marks likely contributed to the fact that RPM calves had greater birth weights (+ 2.8 kg) compared with controls [8]. In newborn animals, Met has been reported to be a limiting AA [165]. On this basis, it is crucial to supplement milk protein-based milk replacers with Met to improve calf performance. Methionine has also been linked with the regulation of monocytes and PMNL in dairy calves. For instance, Abdelmegeid et al. [166] supplied Met to isolated PMNL from neonatal calves and observed a modulatory effect of Met on reducing mRNA abundance of genes involved in inflammation. Furthermore, enhancing Met supply to bovine neonatal hepatocytes decreases mRNA abundance of *BHMT* and *MTR*, suggesting less remethylation of homocysteine to Met [36], which could increase flux of homocysteine through the transsulfuration pathway for antioxidant synthesis.

In terms of growth, calves from RPM-fed cows maintained greater BW and average daily gains throughout 9 weeks of age [9]. Importantly, these changes in growth occurred without changes in intake, indicating that alterations in physiology and metabolism were driving differences in feed efficiency between treatments. One of the factors promoting improved calf growth with maternal RPM supplementation may be the improvement in immune function and antioxidant status. Throughout the preweaning period RPM calves had greater *in vitro* neutrophil phagocytosis than controls, suggesting a priming effect of maternal Met supplementation on calf immune function [97]. We recently reported expression of various AA transporters, AKT and mTOR in PMNL isolated from whole blood. The mRNA abundance of AA transporters changed as calves grew [167], which is likely indicative of the development/maturation of the immune system and the importance of AA. Specifically, there was an increase in solute carrier 43 family A2, a Met transporter, suggesting that Met uptake and its utilization in pathways such as one-carbon metabolism may play an important role in the development of calf immune responses. The detection of AKT and mTOR also suggests that these signaling pathways play a role in regulation of immune function in neonatal calves.

In another study, maternal Met supply during late gestation was associated with changes in mRNA abundance of genes related to inflammation and antioxidant responses in calf PMNL. Specifically, calves born to dams supplemented with RPM had lower expression of *TNF* and myeloperoxidase at birth, while *TLR2*, nitric oxide synthase 2 and cell adhesion molecule 1 were lower throughout the preweaning period [96]. During a whole blood challenge with LPS, a lower basal concentration of IL-1β was also detected in those calves [96], indicating that Met attenuates the proinflammatory response. Concentrations of plasma biomarkers were also altered; ROM, myeloperoxidase and ceruloplasmin concentrations were lower with maternal Met, indicating reduced oxidative stress compared with controls [168]. Maternal Met supply also increased hepatic activity of CBS and the concentration of taurine compared with control calves, suggesting a greater flux of the transsulfuration pathway and a better antioxidant response with maternal Met supply [79]. While more work is needed to understand the mechanisms behind changes in expression of AA transporters and signaling during postnatal growth, these data suggest that Met supply during late pregnancy may alter immune function and oxidative stress to help improve calf health and reduce stress during the neonatal and weaning periods.

#### Choline

Supplying isolated calf PMNL with choline leads to increased mRNA abundance of *GCLC* and decreased *TNF* and *NR3C1* [166]. Although these responses have been detected only at the transcriptomic level and some important molecular markers in the transsulfuration pathway (e.g. *CBS*) have not confirmed similar behavior, the greater *GCLC* abundance expression by choline supplementation could be a mechanism to support greater GSH synthesis. In addition, the downregulation of *TNF* and *NR3C1* supports the hypothesis of alleviation of inflammation in PMNL with greater choline supply [166].

Accumulation of ROS is decreased and VLDL secretion increased when choline is supplied to bovine neonatal hepatocytes, suggesting improvements in antioxidants and liver function [36]. An *in vivo* study reported that choline supply during an endotoxemia challenge with LPS at 4 weeks of age was associated with a reduction in heart and respiratory rates, as well as less severe increase in body temperature [169]. Choline supply in that study also decreased plasma aspartate aminotransferase and prevented an increase in the serum oxidative stress index that was induced by LPS, helping improve liver function and reduce oxidative stress in dairy calves [170].

Investigating the effects of prepartum energy intake on performance of dairy cows supplemented with RPC, Zenobi et al. [171] reported that heifers born from RPC-fed cows experienced greater daily weight gain between birth and the first 50 weeks of age. Although the specific knowledge about the direct effects of this nutrient on immunometabolic and growth regulation in calves is still in its infancy, the combined data underscore potential benefits mainly linked to an increase in antioxidant capacity and hepatic health.

#### Betaine and folic acid

To our knowledge, there is only a single study on the potential nutritional programming effects of betaine supplementation during the periparal period. Calves born from dams fed betaine for the last week of gestation had greater total plasma protein and globulin compared with controls during the first 24 h of life, suggesting an improvement in immunity [104]. Calves from betaine-fed cows also had greater plasma SOD at 2 h after birth, indicating a better antioxidant response [104]. Girard et al. [149] injected cows with FA weekly from 45 d after mating through gestation and followed their calves through 10 weeks of age; FA calves had increased serum folates at birth, but this increase in folates was not associated with differences in birth weight, BW or DMI throughout the experimental period [149].

While more studies are warranted, available data lead us to speculate that enhanced methyl donor supply to dairy calves through the dam’s diet in late pregnancy (epigenetic changes) or directly (milk replacer and/or calf starter) may promote a better basal antioxidant and inflammatory status (e.g. lower pro-inflammatory signaling), which could greatly help these animals that possess an underdeveloped immune system, especially in a period of high-susceptibility to environmental pathogens (i.e. the postnatal stage). Although molecular and cellular mechanisms for antigen recognition and elimination play a critical role in young calf immunity, these mechanisms demand high AA and energetic costs [5, 100, 167]. Because during their first few weeks of life calves need to build a wide variety of innate responses to face pathogens for the first time, regulation of the immune response through anti-inflammatory mechanisms is critical to avoid an immunometabolic overload. Combined data from our research group and others also underscores the importance of methyl donors on calf growth [9, 97, 171]. In fact, nutritional programming strategies have been extensively investigated over the past few decades, and with the advancement of transcriptomic and proteomic methods, a wide field of investigation remains open for future clarification of the role of these nutrients on development, immunity and metabolism of calves.

#### Practical nutritional recommendations for balancing dietary methyl donors

The transition from pregnancy to lactation is a challenging period for dairy cows [1]. In the same way, nutritionists and farmers also face challenges because of the need to focus attention on nutrition and management strategies to avoid metabolic disorders, support optimal DMI, and achieve optimum rates of milk yield. Over the last few decades, our research group and others have reported important production and immunometabolic responses due to the inclusion of methyl donors in the diet, providing critical information for both nutritionists and dairy farmers as they consider balancing rations for methyl donors such as Met and choline [15, 115, 129, 149, 167]. Data indicate that increasing supplementation of methyl donors to dairy cows during the peripartal period often increases performance [10, 106, 129], liver health [28, 33, 62, 130] antioxidant status [19, 172] and immune capacity [90, 92].

Our results from recent studies using different sources of RPM underscored that an optimum ratio of 2.8:1 (Lys:Met) in the metabolizable protein enhances both performance and health during the periparturient period in multiparous Holstein cows [10, 11, 71]. For instance, Osorio et al. [10] supplemented multiparous Holstein cows with two Met sources during the transition period. From − 50 to − 21 d before parturition, cows were fed the same far-off diet (1.24 Mcal/kg of DM, 10.3% RDP, and 4% RUP) without Met supplementation. From − 21 d to expected calving, cows were fed the same close-up diet (1.54 Mcal/kg of DM, 10.0% RDP, and 5.1% RUP), and from calving until 30 DIM, cows were fed the same lactation diet (1.75 Mcal/kg of DM, 10.3% RDP and 4% RUP). Cows were top-dressed with RPM from − 21 to 30 DIM at 0.07% intake of DM. Average Met in metabolizable protein reached 29 g/d and 40 g/d during the prepartum and postpartum periods, respectively. This nutritional management strategy allowed us to observe benefits of RPM on postpartum DMI [10], milk production and composition [10], immune and antioxidant function [10, 33], as well as liver health [89].

Using the same RPM source as Osorio et al. [10], Zhou et al. [71], reported better postpartal performance in multiparous Holstein cows supplemented with RPM compared with RPC during the peripartal period. From − 50 to − 21 d before parturition, cows were fed the same far-off diet (1.40 Mcal/kg of DM, 10.2% RDP, and 4.1% RUP) without Met supplementation. From − 21 d to expected calving, cows were fed the same close-up diet (1.52 Mcal/kg of DM, 9.1% RDP, and 5.4% RUP), and from calving until 30 DIM, the cows were fed the same lactation diet (1.71 Mcal/kg of DM, 9.7% RDP and 7.5% RUP). Cows were top-dressed with RPM from − 21 to 30 DIM at 0.08% intake of DM. Average Met in metabolizable protein reached 33 g/d and 55 g/d during the prepartal and postpartal period, respectively. This strategy was associated with benefits in the plasma AA profile and antioxidant status [18, 172], liver metabolism [18, 35], and immune response [25, 90].

In addition, more recently using a different RPM source, our research group underscored that ethyl-cellulose RPM enhances performance during the peripartal period and early lactation in multiparous Holstein cows [11]. From − 45 to − 28 d before parturition, cows were fed the same far-off diet (1.33 Mcal/kg and 13.9% CP) without Met supplementation. From − 21 d to expected calving, cows were fed the same close-up diet (1.47 Mcal/kg of DM and 15.3% CP). From calving until 30 DIM, cows were fed the same fresh diet (1.67 Mcal/kg of DM and 17.7% CP), and from 31 until 60 DIM, cows were fed the same high-production diet (1.61 Mcal/kg of DM and 17.4% CP). Ethyl-cellulose RPM was supplied from − 28 to 60 d relative to parturition at a rate of 0.09% and 0.10% of DM during the prepartum and postpartum periods, respectively. Based on DMI and the supplementation rates, average Met in the metabolizable protein reached 32 g/d prepartum, 56 g/d during the first 30 DIM and 68 g/d from 31 to 60 DIM. Similar to our previously reported studies, supplementation with RPM, besides promoting increased DMI and milk yield [11], also promoted alleviation of inflammation and oxidative stress [28, 29], coupled with an increase in immune function [29].

As discussed earlier, studies with RPC during the periparturient period have shown that supplemental choline may improve DMI and milk production [128, 129]. Furthermore, there may also be improvements in liver TAG accumulation, liver function and inflammation with enhanced choline supply [37, 38, 101, 130, 131]. However, there is no known requirement for choline in dairy cattle. Regardless, many studies have observed beneficial effects when choline was supplied at 12.5 g/d using RPC, suggesting that this may be the optimal dose. When choline was supplied at 0, 6.25, 12.5 and 25 g/d via abomasal infusion during a feed restriction-induced NNB (60% net energy for lactation), 12.5 g/d had the greatest improvement in fat-corrected milk and greatest reduction in liver TAG [38], thus, supporting the data from the meta analyses.

From a practical standpoint, it is also important to note that recent research has indicated that the effects of supplying choline may vary depending on age, i.e. between primi- and multiparous cows. For example, Potts et al. [173] supplemented cows with RPC (13 g/d), RPM (13.5 g/d) or RPM and RPC for 3 weeks prepartum and 5 weeks postpartum and observed that primiparous cows receiving RPC had increased milk yields irrespective of RPM supply; however, multiparous cows only had an increase in milk yields when RPC was supplemented alone [173]. These responses could be due to differences in nutrient needs between primiparous and multiparous cows. Bollatti et al. [174] observed that timing of choline supplementation also may be important. They used a 2 × 2 factorial arrangement of treatments to supply 0 or 12.9 g/d choline using RPC during the transition period (21 d pre- and postpartum), and this was followed by supplementation of 0 or 12.9 g/d choline during the post-transition period (d 22 through 105 postpartum). They observed that choline supply during the transition period increased milk fat percentage and yield, and energy-corrected milk [174]; there was also a carryover effect of RPC supplementation during the transition period, with those cows having increased milk and energy-corrected milk yields, as well as increased milk fat yields during the post-transition period [174]. However, there were no effects on production when RPC was supplied during the post-transition period [174]. Lastly, it is important to note that Bollatti et al. [175] reported that improvements in milk, fat and protein yields, as well as increases in energy- and fat-corrected milk with RPC supply during the peripartal period were not dependent on prepartum BCS. Overall, the above studies suggest that enhanced choline supply will be most-beneficial during the peripartal period, and that it may be particularly beneficial for primiparous cows.

Regarding FA and B_12_ supplementation in peripartual dairy cows, the literature underscores that responses to both nutrients are directly related to the status of these vitamins in the animal [155]. When plasma B_12_ concentrations are lower than ~ 200 pg/mL, the cellular use of FA can be impaired, as well as when plasma FA concentrations are lower than ~ 12 ng/mL, the transfer of B_12_ from the cytosol to mitochondria could be impaired [106, 149, 150]. Because the routine measurement of plasma FA and B_12_ profile is impractical in commercial farms, supplementation strategies can play an important role in the balance of these nutrients within the cow. Increases in plasma FA concentrations were positively related to diets with high levels of nonfiber carbohydrates and negatively associated with fiber-rich diets, while plasma B_12_ concentrations had an opposite behavior, i.e. positively related to fiber-rich diets and negatively associated with diets rich in nonfiber carbohydrates [176, 177].

Available studies underscored that supplementing both vitamins together can promote important benefits on milk production and composition through changes in energy partitioning without changes in DMI [106, 107]. It appears that this energy support is beneficial to mechanisms of fat mobilization, with lower body weight loss observed in supplemented lactating cows [151, 178, 179]. For instance, Preynat et al. [156] investigated the effects of weekly intramuscular injections of 160 mg of FA plus 10 mg of B_12_ from 3 weeks before parturition until 12 weeks of lactation in diets with different Met levels. Supplemental FA and B_12_ led to an increase in milk production, lactose, protein, and total solids yields with no differences in DMI. These responses were partly explained due to a greater whole-body glucose flux in vitamin-supplemented cows. This lent support to the idea of benefits in energy partitioning during early lactation [156]. Using a different approach, Graulet et al. [106] evaluated the effects of dietary supplements of FA and B_12_ from 3 weeks before to 8 weeks after parturition. Supplemental FA at 2.6 g/d and B_12_ at 0.5 g/d were top-dressed with the morning meal. This study reported increases in milk and milk protein yields due to FA, regardless of B_12_ supply. However, the combined supplementation of both vitamins seemed to improve metabolic efficiency, glucose availability, and liver health [106].

In a larger study with 805 dairy cows from 15 commercial farms, Duplessis et al. [178] investigated the effects of weekly intramuscular injections of 320 mg of FA plus 10 mg of B_12_ given 21 d before parturition until 60 DIM. During the first 60 DIM there were no effects on average milk yield due to treatments, or milk production during the 305 d of lactation. Authors reported a decrease in milk fat and an increase in milk protein leading to a lower fat:protein ratio due to FA and B_12_ supply. Those responses coupled with reduced loss of estimated body weight after parturition in treated cows suggest that these supplementation strategies also changed energy partitioning during early lactation as reported by Preynat et al. [107]. Thus, it is noteworthy that responses due to FA and B_12_ supplementation are directly related to the status of both vitamins in the cow. Although studies reported benefits mainly during early lactation, some inconsistencies in data repeatability suggest that the mechanisms described above for the increased milk yield due to improvements in energy partitioning deserve further investigation.

## Conclusions

Methyl donors serve as functional nutrients whose role in regulating key metabolic and immunological pathways stems from one-carbon metabolism. These unique functions make modulation of dietary methyl donors during periods of stress, e.g. neonatal and peripartal periods, a viable option for improving production and health in dairy cows and calves. The data supporting the use of supplemental methyl donors to enhance metabolism and health in peripartal cows are strong, particularly for Met and choline. Furthermore, modulation of prepartal adiposity and pasture allowance appear to be feasible strategies to obtain similar results in pasture-based systems, where direct supplementation of methyl donors might not be possible or practical. However, there is a paucity of information on molecular mechanisms by which methyl donors elicit these effects in dairy cattle. Understanding better these mechanisms and their benefits for ruminants will be enhanced by the application of a systems biology approach, i.e. combine production data with high-throughput ‘omics’ techniques.

### Prospective opportunities for the future

The development of ruminal “protection” technologies for methyl donors will also be key to furthering methyl donor research. Interest in nutritional programming during pregnancy is continuing to grow in the dairy industry. Alterations in maternal dietary methyl donor supply, particularly Met, have been shown to modulate calf growth, metabolism and immune responses during the pre-weaning period. However, more work is needed on the effects of other methyl donors, as well as on the potential long-term effects on future milk production. While more work is needed, based on the available data it is clear that methyl donor supply during the peripartal period is key to promoting optimal immunometabolism of cows and calves. By improving immunometabolism during this period, health and productivity will be improved, leading to more efficient dairy production. Thus, the integration of nutritional programming research with peripartal cow research will be key in the future as we seek to fine-tune diets for optimizing efficiency in both cows and calves.

## Data Availability

Not available.

## Abbreviations

AA: Amino acid
AKT: Protein kinase B
BCS: Body condition score
BHMT: Betaine homocysteine methyltransferase
BW: Body weight
CBS: Cystathionine β-synthase
DMI: Dry matter intake
DNMT: DNA methyltransferase
GCLC: Glutamate-cysteine ligase catalytic subunit
GSH: Glutathione
GPX: Glutathione peroxidase
LPS: Lipopolysaccharide
MAT: Methionine adenosyltransferase
Met: Methionine
mTOR: Mechanistic target of rapamycin
mTORC1: mTOR complex 1
MTR: 5-Methyltetrahydrofolate-homocysteine methyltransferase
NFE2L2: Nuclear factor erythroid 2-like-2
NNB: Negative nutrient balance
P: Phosphorylated
PC: Phosphatidylcholine
PMET: Phosphatidylethanolamine methyltransferase
PMNL: Polymorphonuclear leukocytes
PPARA: Peroxisomal proliferator activated receptor α
RPC: Rumen-protected choline
RPM: Rumen-protected methionine
SAH: *S*-Adenosylhomocysteine
SAHH: *S*-Adenosylhomocysteine hydrolase
SAM: *S*-Adenosyl methionine
SAMTOR: *S*-Adenosylmethionine sensor upstream of mTOR
SAT: Subcutaneous adipose tissue
SLC22A5: Solute carrier family 22 member A5
SOD1: Superoxide dismutase 1
TAG: Triacylglycerol
TLR: Toll-like receptor
TMR: Total mixed ration
TNF- α: Tumor necrosis factor-α
VLDL: Very low-density lipoprotein
